# Impact of physical activity on caloric and macronutrient intake in children and adolescents: a systematic review and meta-analysis of randomized controlled trials

**DOI:** 10.1186/s12966-024-01620-8

**Published:** 2024-07-15

**Authors:** Heiko Hahn, Manuel Friedel, Claudia Niessner, Stephan Zipfel, Isabelle Mack

**Affiliations:** 1 Department of Psychosomatic Medicine and Psychotherapy, University Medical Hospital Tübingen, Osianderstr. 5, Tübingen, 72076 Germany; 2 Institute of Sports and Sport Science, Karlsruhe Institute of Technology, Engler-Bunte-Ring 15, Karlsruhe, 76131 Germany

**Keywords:** Energy intake, Exercise, Physical activity, Children and adolescents

## Abstract

**Background:**

Physical activity is widely promoted to maintain and improve health across all ages. Investigating how physical activity affects subsequent food intake provides insight into the factors that contribute to maintaining energy balance and effective weight management.

**Objective:**

This systematic review and meta-analysis summarizes the evidence on the effect of acute physical activity on subsequent food intake in children and adolescents.

**Methods:**

The Preferred Reporting Items for Systematic Reviews and Meta-Analyses guidelines (PRISMA) were applied. Randomized controlled trials (RCTs) objectively measuring post-exercise energy intake in children and adolescents aged 5 to 18 years were included. Studies with self-reported food intake were excluded. The databases PubMed, Web of Science and Cochrane Library were searched for RCTs, and the data were summarized at a qualitative and quantitative level. Version 2 of the Cochrane risk-of-bias tool for randomized trials was used to assess risk of bias. Changes in energy intake were examined with random effects meta-analysis. (PROSPERO: CRD42022324259).

**Results:**

Out of 9582 studies, 22 RCTs with cross-over design remained eligible for meta-analysis. The primary outcome was post-intervention energy intake up to the next 24 h. Heterogeneity of studies was moderate, with an I^2^ of 57%. The median (interquartile range, IQR) energy expended while exercising was 240 (158) kcal. Meta-analysis of 41 study arms (exercise *n* = 780 and control *n* = 478) showed no differences in total energy intake between the exercise and control group with a mean difference MD = 23.31 [-27.54, 74.15] kcal. No subgroup differences were found. Macronutrient intake and appetite sensations where not substantially affected.

**Conclusion:**

Engaging in exercise is a suitable means of raising activity-induced energy expenditure, without causing any noticeable changes in food intake or hunger within a single day.

**Supplementary Information:**

The online version contains supplementary material available at 10.1186/s12966-024-01620-8.

## Introduction

Energy balance is regulated by a complex interplay between energy intake and energy expenditure [1, 2]. When the balance tilts in favor of energy intake, it leads to obesity, a significant public health concern from both personal and socio-economic perspectives [3]. Although rising trends in child and adolescent body mass index (BMI) have plateaued at high levels in many high-income countries, they have accelerated in parts of Asia [4]. This trend is attributed to a combination of factors such as decreased physical activity, increased sedentary behavior [5] and the overconsumption of high-energy-dense foods and large food portions [6, 7]. Decades of research have provided a comprehensive understanding of the factors influencing energy balance [8–11], resulting in similar recommendations for daily physical activity and a healthy diet globally [12–14]. However, adherence to these recommendations is challenging, as evidenced by alarming obesity rates [15]. Many research questions remain, particularly concerning body weight maintenance [16, 17]. Hence, ongoing research aims to comprehend the complex interplay of factors contributing to energy balance to offer holistic recommendations to society and patients while identifying targets for medication and interventions.

Food intake is influenced by environmental, psychological, and physiological factors [18]. The primary physiological driver of food intake is the resting metabolic rate (RMR), with fat-free mass being its largest contributor [19, 20]. RMR constitutes the largest component of daily energy expenditure and remains relatively stable throughout the day, generating a constant energetic demand [21, 22]. In contrast, acute exercise creates a short-term high energy requirement and induces various physiological effects as a result of increased sympathetic activity [23, 24].

Due to the distinct nature of these predictors of food intake, they are expected to have different mechanistic effects on appetite control, as summarized by Blundell et al. [25]. While RMR is relatively stable and cannot be acutely altered, physical activity can significantly increase energy expenditure through muscle activity [26, 27]. Activity energy expenditure is a crucial determinant of energy intake [28, 29]. Thus, exercise can facilitate weight loss by boosting energy expenditure, although this effect can be counteracted by subsequent sedentary behavior and increased food intake [30].

Structured exercise programs for adults with obesity have shown varying outcomes regarding body weight changes, ranging from weight loss to weight gain [31, 32]. This variability indicates that the determinants of sedentary behavior and post-exercise food intake are not yet fully understood. Physical activity may stimulate appetite to compensate for burned energy, but it can also lead to decreased appetite and increased sensitivity to satiety signals [33–35].

While the acute (up to 24 h) and short-term (up to 14 days) effects of physical activity on food intake in adults have been extensively studied [36–42], data for children and adolescents are limited [43]. The most recent systematic review on this topic was conducted in 2016 by Thivel et al. [44], concluding that acute exercise did not affect energy intake in lean individuals but appeared to reduce food intake in youth with obesity when exercise intensity was high. Since then, further high-quality trials have been published, allowing for a more robust analysis of data with stricter inclusion criteria, which is the focus of this review.

The aim of this review was to analyze the effects of acute exercise on energy intake under controlled conditions in children and adolescents. The main research questions were: i) whether acute exercise leads to increased food intake under controlled conditions; ii) whether these findings depend on the intensity, duration, and type of exercise; and iii) whether these findings are independent of body weight status. Understanding these fundamental aspects under controlled conditions is crucial for advancing basic knowledge within the broader context of energy balance regulation, contributing to the development of tailored public health strategies.

## Materials and Methods

### Literature information sources and search strategy

This review was developed and executed according to the Preferred Reporting Items for Systematic Reviews and Meta-Analyses (PRISMA) guidelines [45]. To identify all relevant studies examining the effect of physical activity on caloric and macronutrient intake in children and adolescents across all weight categories, the databases PubMed, Web of Science and Cochrane Library were searched on November 16th and November 20th, 2021. The protocol of this systematic review is registered at the PROSPERO platform with the registration number CRD42022324259. The full search strategy is documented in the Supporting Information Text S1 and consisted of four modules in the search term: children and adolescents, physical activity, energy intake and macronutrient intake.

### Eligibility Criteria

Eligibility criteria were based on the five PICOS dimensions, i.e., participants (P), interventions (I), comparators (C), outcome (O) and study design (S) [46]. Only peer-reviewed original studies written in English or German were included.

*Participants:* Participants included healthy, non-smoking children and adolescents aged on average ≥ 5 year to ≤ 18 years old, without any restrictions on sex, ethnicity, and weight status.

*Interventions:* Physical exercise intervention had to be conducted under guidance and supervised conditions without restrictions regarding intensity, duration, and modality. Recording of vital signs was not a prerequisite. The exercise intervention had to be followed by at least one post-exercise ad libitum meal under controlled conditions, such as a laboratory or researcher-controlled setting without restrictions regarding the characteristics of the meal conditions (e.g., ad libitum buffets, ad libitum single or multiple meals). Trials that additionally implemented dietary interventions or nutritional education were excluded.

*Comparators:* A comparison to controls was required, either between or within subjects.

*Outcomes:* The primary outcome was energy intake in kcal resulting from the corresponding food intake after the exercise intervention. Therefore, food intake in grams had to be measured by a calibrated scale and the foods’ caloric value had to be derived from validated sources, either bomb calorimetry or internationally known food databases. Data from food frequency questionnaires, 24-h recalls, or similar sources were excluded. Secondary outcomes were food intake (in grams) and macronutrient intake (in grams and as % energy intake) along with the appetite sensations hunger, satiety, and prospective food consumption (i.e. how much food participants thought they could eat).

*Study designs*: The systematic data analysis referred exclusively to randomized controlled trials as parallel and crossover designs.

### Study selection, data collection and organisation

To identify eligible studies, the search results of the databases were combined, and the duplicates removed. Next, the titles and abstracts were screened. Full-text articles were evaluated regarding their eligibility (HH and IM), with uncertainties being discussed between the authors (< 15%). In the case of discrepancies, a third author was involved (MF).

### Data items and statistics

The following information was extracted from each included article: year of publication, country of origin, study type, type of intervention, method for data collection, study outcomes including caloric intake, macronutrient intake and appetite sensations, and sample characteristics (including sample size, BMI, sex, and age).

Characteristics across studies are presented as absolute values for sample size, sex and exercise duration, as mean and standard deviation (SD) or per cent (%) for macronutrient intake and exercise intensity and as mean and SD or standard error (SE) for sample size, age, BMI, energy expenditure and energy intake. All energy intake and energy expenditure values were converted to kilocalories (kcal).

Exercise intensity was determined to be either low, moderate, or high, based on percentages of maximum oxygen consumption (VO2max) or maximum heart rate (HRmax) [47, 48]. Intensity was low if average heart rate was < 64% of HRmax or if VO2max was < 50%. Intensity was moderate if average heart rate was ≥ 64%—≤ 76% of HRmax, or if VO2max was ≥ 50%—< 70%, or if exercise was performed at ventilation threshold. Intensity was high if average heart rate was ≥ 77% of HRmax or if VO2max was ≥ 70%. In studies in which neither heart rate nor VO2 max was measured, the classification into low, moderate, and high was adopted according to the classification given in the trials.

For the energy intake (kcal), the results of all 22 trials were evaluated quantitatively (meta-analysis) and qualitatively. Qualitative analysis was also carried out to describe the direction of change in energy intake (whether participants ate more, the same or less) between exercise and control groups. In the case of missing data, the provided graphs were measured with the help of a software tool (Digitizeit [49] and WebPlotDigitizer [50]) to obtain the values needed. For the meta-analysis of cross-over trials, the mean difference (MD) and standard error (SE) were calculated according to the Cochrane Handbook for Systematic Reviews of Interventions [51]. If the studies did not provide sufficient data from a paired analysis, a correlation coefficient of 0.5 was set. This approach is consistent with that of another meta-analysis of exercise interventions [52], and is described in detail by Elbourne et al. [53]. Sensitivity analyses were performed for correlation coefficients of 0.3 and 0.7. The results were then entered into a generic inverse variance approach with the random-effects model using the software package Review Manager, version 5.4 [54]. In studies with multiple intervention arms the sample size of the shared group was split according to the Cochrane Handbook [51] and Rücker et al. [55] to avoid “double-counting” of participants (unit-of-analysis error). For the meta-analysis, 41 study arms were eligible. The difference in energy intake in kcal is presented as mean difference (MD), 95% confidence interval (CI) and standard error (SE) and is displayed in forest plots.

Statistical heterogeneity was examined by visual inspection of forest plots and using the I^2^ statistics to quantify inconsistency between the studies. Values < 25% were interpreted as low, 25%—75% as moderate and values > 75% were interpreted as high [56]. To reduce heterogeneity, subgroup analyses were performed for intensity of physical activity, age, risk of bias and weight status.

Data on the appetite sensations hunger, satiety, and prospective food consumption was evaluated qualitatively because different assessment tools were used, and many studies did not report data but only stated that either differences or no differences were found between the groups. Authors were contacted in case of missing data up to three times and 33% (2 out of 6) responded to the inquiry.

### Risk of Bias

For all eligible studies, a risk of bias assessment was conducted using the Cochrane Risk-of-Bias tool for randomized crossover trials (RoB 2) [57]. The tool consists of 5 domains addressing different types of bias: randomization process, deviations from the intended interventions, missing outcome data, measurement of the outcome and selection of the reported result. In each domain, appropriate questions must be answered for each single study. Next, the RoB 2 algorithm is applied which evaluates the risks of the individual domains. Finally, an overall risk is calculated and expressed as “low” or “high” risk of bias, or the judgment can be expressed with “some concerns”.

## Results

### Study selection and categorization

**Fig. 1:**
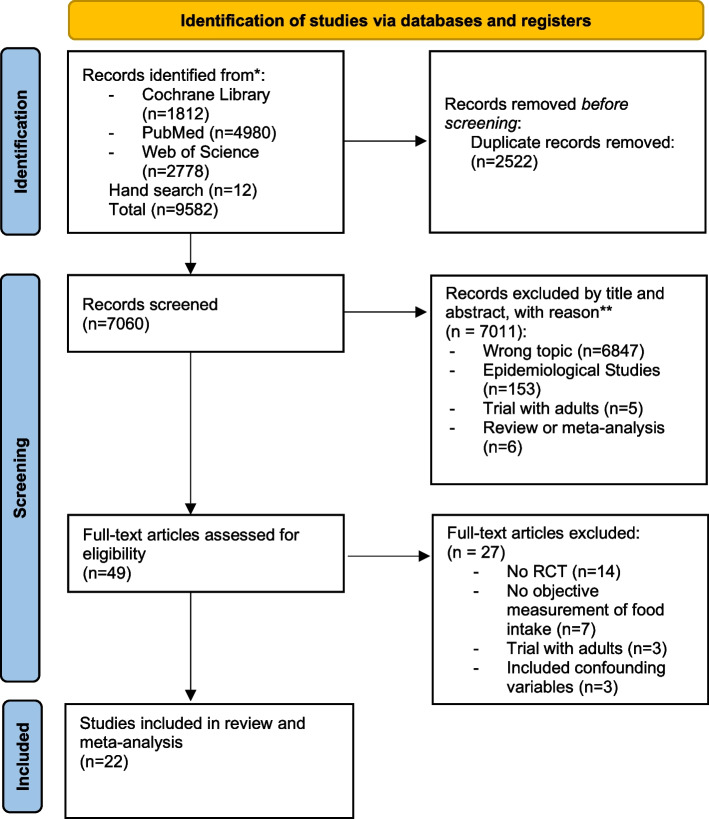
PRISMA flow chart for study inclusion

The literature search process used to identify eligible studies is shown in Fig. 1. Out of 9582 identified studies, 22 studies remained for analysis.

### Summary of study characteristics

A detailed overview of the characteristics of the individual trials is presented in Table 1. The characteristics across the studies are given below.

**Table 1:** Overview of characteristics of trials

Study	Population	Exercise characteristics	Meal characteristics	Absolute Energy Intake in kcal (mean ± SD) Statistically significant change in intake (↑, ↓, ↔)	Macronutrients Statistically significant change in Intake (↑, ↓, ↔)
Ajibewa et al. 2017 [58]	n: 26 (NR) Age: 7 – 11 y BMI: normal weight, not further specified.	Modality: Static stretching, standing and yoga poses Duration: 20 x 2 min Intensity: Resting heart rate + heart rate reserve * 25% EE: NR	Pre-Intervention: Three standardized meals Post-Intervention: One meal ad libitum	CON: 999 ± 62 (SE) EX: 982 ± 50 (SE) ↔	NR
		Modality: Push-ups, sit-ups, and age-appropriate calisthenics Duration: 20 x 2 min Intensity: Resting heart rate + heart rate reserve * 50% EE: NR		CON: 999 ± 62 (SE) EX: 926 ± 63 (SE) ↔	
		Modality: Vigorous calisthenics (e.g., jumping jacks) Duration: 20 x 2 min Intensity: Resting heart rate + heart rate reserve * 75% EE: NR		CON: 999 ± 62 (SE) EX: 1016 ± 76 (SE) ↔	
	n: 13 (NR) Age: 7 – 11 y BMI: obese, not further specified.	Modality: Static stretching, standing and yoga poses Duration: 20 x 2 min Intensity: Resting heart rate + heart rate reserve * 25% EE: NR		CON: 1145 ± 88 (SE) EX: 1204 ± 71 (SE) ↔	
		Modality: Push-ups, sit-ups, and age-appropriate calisthenics Duration: 20 x 2 min Intensity: Resting heart rate + heart rate reserve * 50% EE: NR		CON: 1145 ± 88 (SE) EX: 1066 ± 89 (SE) ↔	
		Modality: Vigorous calisthenics (e.g., jumping jacks) Duration: 20 x 2 min Intensity: Resting heart rate + heart rate reserve * 75% EE: NR		CON: 1145 ± 88 (SE) EX: 1261 ± 103 (SE) ↔	
Bozinovski et al. 2009 [59]	n: 29 (14 m, 15 f) Age: 12.1 ± 0.4 y BMI: 54.3rd ± 5.3 percentile	Modality: Treadmill Duration: 15 min Intensity: Ventilation Threshold EE: 63 ± 7 kcal	Pre-Intervention: Standardized breakfast Post-intervention: 250mL water, pizza meal ad libitum 30 min post-exercise	CON: 1078 ± 101 (SE) EX: 1060 ± 103 (SE) ↔	NR
		Modality: Treadmill Duration: 45 min Intensity: Ventilation Threshold EE: 189 ± 14 kcal		CON: 1078 ± 101 (SE) EX: 1101 ± 92 (SE) ↔	
Fearnbach et al. 2016 [60]	n: 15 m Age: 13.8 ± 1.5 y BMI: 31.8 ± 4.2 kg/m²	Modality: Cycling Duration: 45 min Intensity: 65% VO2max EE: 399 ± 75 kcal	Pre-Intervention: Standardized breakfast	CON: 1116 ± 243 EX: 1037 ± 260 ↓	Protein (%) CON: 29.4 ± 7.2 EX: 30.5 ± 6.7 ↔
			Post-Intervention: Buffet meal ad libitum 30 min post-exercise		Fat (%) CON: 16.5 ± 4.2 EX: 16.6 ± 4.2 ↔
					CHO (%) CON: 53.4 ± 11.0 EX: 52.3 ± 10.5 ↔
Fearnbach, Masterson et al. 2016 [61]	n: 20 (12 m, 8 f) Age: 10.3 ± 1.1 y BMI: 41.6 ± 21.7 percentile	Modality: Cycling Duration: 30 min Intensity: 70% VO2max EE: 534 ± 263 kcal	Pre-Intervention: Standardized breakfast	CON: 2088 ± 497 EX: 2171 ± 566 ↔	Protein (kcal) CON: 196 ± 67 EX: 207 ± 68 ↑
			Post-Intervention: Standardized snack; lunch and dinner meal ad libitum, timing not reported		Fat (kcal) CON: 655 ± 155 EX: 694 ± 181 ↑
					CHO (kcal) CON: 1235 ± 295 EX: 1269 ± 337 ↔
Fearnbach, Silvert et al. 2017 [62]	n: 14 m Age: 13.9 ± 1.1 y BMI: 31.6 ± 4.5 kg/m²	Modality: Cycling Duration: 45 min Intensity: 65% VO2max EE: 373 ± 57 kcal	Pre-Intervention: Standardized breakfast	CON: 1091 ± 252 EX: 965 ± 214 ↓	Protein (%) CON: 31.1 ± 5.5 EX: 31.8 ± 4.8 ↔
			Post-Intervention: Buffet meal ad libitum 30 min post-exercise		Fat (%) CON: 17.5 ± 3.2 EX: 17.1 ± 3.3 ↔
					CHO (%) CON: 50.8 ± 8.3 EX: 50.4 ± 7.7 ↔
	n: 14 m Age: 13.7 ± 1.1 y BMI: 19.2 ± 3.2 kg/m²	Modality: Cycling Duration: 45 min Intensity: 65% VO2max EE: 241 ± 93 kcal		CON: 854 ± 362 EX: 744 ± 246 ↔	Protein (%) CON:28.0 ± 7.2 EX: 28.1 ± 5.3 ↔
					Fat (%) CON: 16.4 ± 4.9 EX: 16.1 ± 4 ↔
					CHO (%) CON: 55.1 ± 11.1 EX: 52.2 ± 8.6 ↔
Fillon et al. 2020 [63]	n: 18 (12 m, 6 f) Age: 12.7 ± 1.3 y BMI: 33.3 ± 6.5 kg/m²	Modality: Cycling Duration: 30 min Intensity: 65% VO2max EE: 169 ± 44 kcal	Pre-Intervention: Standardized breakfast	CON: 2175 ± 330 EX: 2277 ± 476 ↔	Protein (%) CON: 21.3 ± 2.5 EX: 21.0 ± 2.0 ↔
			Post-Intervention: Buffet lunch meal ad libitum 30 min post- exercise; dinner buffet meal ad libitum		Fat (%) CON: 30.7 ± 5.8 EX: 31.2 ± 4.8 ↔
					CHO (%) CON: 47.8 ± 7.4 EX: 47.4 ± 6.1 ↔
			Pre-Intervention: Standardized breakfast	CON: 2175 ± 330 EX: 1925 ± 360 ↓	Protein (%) CON: 21.3 ± 2.5 EX: 20.6 ± 2.3 ↔
					Fat (%) CON: 30.7 ± 5.8 EX: 30.5 ± 5.7 ↔
			Post-Intervention: Buffet lunch meal ad libitum 90 min post- exercise; dinner buffet meal ad libitum		CHO (%) CON: 47.8 ± 7.4 EX: 48.7 ± 7.3 ↔
Fillon, Beaulieu et al. 2020 [64]	n: 17 (9 m, 8 f) Age: 12.8 ± 1.4 y BMI: 33.4 ± 5.7 kg/m²	Modality: Cycling Duration: 30 min Intensity: 65% VO2max EE: 135 kcal ± NR	Pre-Intervention: Not clearly reported Post-Intervention: Lunch ad libitum immediately post- exercise; dinner buffet ad libitum	CON: 1997 ± 514 EX: 1939 ± 501 ↔	Protein (%) CON: NR EX: NR ↔
					Fat (%) CON: NR EX: NR ↔
					CHO (%) CON: NR EX: NR ↔
Fillon, Mathieu et al. 2020 [65]	n: 15 (6 m, 9 f) Age: 13.1 ± 1.4 y BMI: 34.7 ± 6.0 kg/m²	Modality: Cycling Duration: 30 min Intensity: 65% VO2max EE: 186 ± 52 kcal	Pre-Intervention: Standardized breakfast	CON: 2004 ± 430 EX: 1948 ± 416 ↔	Protein (%) CON: 22.0 ± 2.5 EX: 24.1 ± 3.7 ↔
			Post-Intervention:Lunch meal ad libitum180 min post-exercise;dinner buffet meal adlibitum		Fat (%) CON: 30.8 ± 4.8 EX: 27.1 ± 7.0 ↔
					CHO (%) CON: 46.9 ± 6.4 EX: 48.7 ± 8.9 ↔
			Pre-Intervention: Standardized breakfast	CON: 2004 ± 430 EX: 1820 ± 459↔	Protein (%) CON: 22.0 ± 2.5 EX: 23.5 ± 3.7 ↑
			Post-Intervention: Lunch meal ad libitum 60 min post-exercise; dinner buffet meal ad libitum		Fat (%) CON: 30.8 ± 4.8 EX: 26.7 ± 8.1 ↔
					CHO (%) CON: 46.9 ± 6.4 EX: 49.0 ± 10.5 ↔
Masurier et al. 2018 [66]	n: 20 f Age: 13.3 ± 1.0 y BMI: 31.6 ± 3.9 kg/m²	Modality: cycling Duration: 20 min Intensity: Ventilation Threshold (54.1 ± 5.4% of VO2max) EE: 117 ± 22 kcal	Pre-Intervention: Standardized breakfast	CON: 738 ± 320 EX: 854 ± 450 ↔	Protein (%) CON: 16.3 ± 4.2 EX: 18.2 ± 4.5 ↔
			Post-Intervention: Buffet meal ad libitum 30 min post-exercise		Fat (%) CON: 9.7 ± 2.6 EX: 11.5 ± 11.5 ↔
					CHO (%) CON: 71.1 ± 13.1 EX: 69.9 ± 7.4 ↔
		Modality: Cycling Duration: 40 min Intensity: Ventilation Threshold (54.1 ± 5.4% of VO2max) EE: 235 ± 44 kcal		CON: 738 ± 320 EX: 806 ± 375 ↔	Protein (%) CON: 16.3 ± 4.2 EX: 17.5 ± 3.2 ↔
					Fat (%) CON: 9.7 ± 2.6 EX: 11.1 ± 2.7 ↔
					CHO (%) CON: 71.1 ± 13.1 EX: 71.0 ± 5.4 ↔
Miguet et al. 2018 [67]	n: 33 (12 m, 21 f) Age: 13.0 ± 0.9 y BMI: 35 ± 4.3 kg/m²	Modality: Cycling Duration: 15 min (5x 2 min high, 30 sec. Low intensity) Intensity: High intensity intervals EE: 102 ± 21 kcal	Pre-Intervention: Standardized breakfast	CON: 2177 ± 471 EX: 2062 ± 460 ↓	Protein (%) CON: 22.6 ± 3.4 EX: 22.7 ± 3.3 ↔
			Post-Intervention: Lunch buffet ad libitum 30 min post-exercise; dinner buffet ad libitum		Fat (%) CON: 32.7 ± 6.1 EX: 31.9 ± 5.9 ↔
					CHO (%) CON: 45.3 ± 7.1 EX: 46.1 ± 7.03 ↔
Morris et al. 2018 [68]	n: 10 (5 m, 5 f) Age: 9.8 ± 0.6 y BMI: 18.3 ± 2.6 kg/m²	Modality: Sprints Duration: 22 min (8 x 30 sec) Intensity: high intensity intervals EE: NR	Pre-Intervention: Same breakfast on both experimental days	CON: 500 ± 69 EX: 492 ± 84 ↔	Protein (g) CON: 12.7 ± 1.2 EX: 12.5 ± 1.5 ↔
			Post-Intervention: Lunch meal ad libitum 5-10 min post-exercise		Fat (g) CON: 23.4 ± 3.2 EX: 22.9 ± 3.7 ↔
					CHO (g) CON: 60.2 ± 9.5 EX: 58.4 ± 11.6 ↔
Nemet et al. 2010 [69]	n: 22 (7 m, 15 f) Age: 9.1 ± 0.6 y BMI: 23.9 ± 0.6 kg/m^2^	Modality: Aerobic games Duration: 45 min Intensity: high EE: 9.6 kcal / kg Bodyweight (BW)	Pre-Intervention: Controlled diet 24h prior to experimental days	CON: 806 ± 51 (SE) EX: 935 ± 81 (SE) ↑	NR
		Modality: Swimming Duration: 45 min Intensity: moderate EE: 7.6 kcal / kg BW	Post-Intervention: Lunch buffet ad libitum 30–45 min post-exercise	CON: 806 ± 51 (SE) EX: 990 ± 106 (SE) ↑	
		Modality: Resistance Duration: 45 min Intensity: moderate EE: 6.3 kcal / kg BW		CON: 806 ± 51 (SE) EX: 779 ± 84 (SE) ↔	
	n: 22 (5 m, 17 f) Age: 9.4 ± 0.3 y BMI: 17.0 ± 0.4 kg/m²	Modality: Aerobic games Duration: 45 min Intensity: high EE: 10.2 kcal / kg BW		CON: 604.7 ± 64.5 (SE) EX: 579.3 ± 34.1 (SE) ↔	
		Modality: Swimming Duration: 45 min Intensity: moderate EE: 8.1 kcal / kg BW		CON: 604.7 ± 64.5 (SE) EX: 484.9 ± 44.4 (SE) ↔	
		Modality: Resistance Duration: 45 min Intensity: moderate EE: 6.9 kcal / kg BW		CON: 604.7 ± 64.5 (SE) EX: 435.9 ± 41.8 (SE) ↓	
Saunders et al. 2013 [70]	n: 20 (8 m, 12 f) Age: 12.2 ± 0.9 y BMI: 18.6 ± 4.3 kg/m²	Modality: Walking Duration: 2 min every 20 min (42 min total) Intensity: low EE: 744 ± 141 kcal (in 9h)	Pre-Intervention: Standardized breakfast	CON: 1176 ± 459 EX: 1218 ± 467 ↔	Protein (%) CON: 10.68 ± 2.51 EX: 11.46 ± 3.32 ↔
			Post-Intervention: Standardized lunch; dinner buffet ad libitum 3h post-exercise		Fat (%) CON: 34.51 ± 7.3 EX: 33.3 ± 8,1 ↔
					CHO (%) CON: 54.81 ± 7.6 EX: 55.24 ± 9 ↔
		Modality: Walking + Treadmill Duration: 2 min every 20 min (42 min total) + 40 min treadmill Intensity: 20 min at 60% VO2max + 20 min at 30% VO2max EE: 970 ± 219 kcal (in 9h)		CON: 1176 ± 459 EX: 1265 ± 503 ↔	Protein (%) CON: 10.68 ± 2.51 EX: 10.71 ± 3.13 ↔
					Fat (%) CON: 34.51 ± 7.3 EX: 35.61 ± 9.38 ↔
					CHO (%) CON: 54.81 ± 7.6 EX: 53.7 ± 9.1 ↔
Thivel, Isacco, Rousset et al. 2011 [71]	n: 12 (5 m, 7 f) Age: 14.4 ± 1.5 y BMI: 35.1 ± 7.6 kg/m²	Modality: Cycling Duration: 30 min Intensity: 70% VO2max EE: 298 ± 28 kcal	Pre-Intervention: Standardized breakfast	CON: 2214 ± 222 EX: 1935 ± 220 ↓	Protein (kcal) CON: 192 ± 33 EX: 206 ± 42 NR
			Post-Intervention: Lunch buffet ad libitum 30 min post-exercise; dinner buffet ad libitum		Fat (kcal) CON: 327 ± 66 EX: 373 ± 64 NR
					CHO (kcal) CON: 453 ± 120 EX: 367 ± 76 NR
Thivel, Isacco, Taillardat et al. 2011 [72]	n: 14 (7 m, 7 f) Age: 14.1 ± 1.8 y BMI: 33.9 ± 7.5 kg/m²	Modality: Cycling Duration: 3 x 10 min (2 min rest in between) Intensity: 70% VO2max EE: 299 ± 29 kcal	Pre-Intervention: Standardized breakfast Post-Intervention: Lunch buffet ad libitum 30 min post-exercise; dinner buffet ad libitum	CON: 1808 ± 301 EX: 1576 ± 394 ↓	NR
Thivel et al. 2012 [73]	n: 15 m Age: 13.5 ± 0.9 y BMI: 30.7 ± 4.1 kg/m²	Modality: Cycling Duration: 59 ± 6 min Intensity: 40% VO2max EE: 336 ± 50 kcal	Pre-Intervention: Calibrated breakfast	CON: 3620 ± 694 EX: 3820 ± 584 ↔	Protein (%) CON: 20.72 ± 4.69 EX: 19.5 ± 3.21 ↔
			Post-Intervention: Lunch buffet ad libitum 30 min post-exercise; dinner buffet ad libitum; breakfast buffet ad libitum the next morning		Fat (%) CON: 20.72 ± 4.69 EX: 43.44 ± 9.58 ↔
					CHO (%) CON: 34.22 ± 8.73 EX: 37.04 ± 10.36 ↔
		Modality: CyclingDuration: 30 ± 3 minIntensity: 75% VO2maxEE: 332 ± 47 kcal		CON: 3620 ± 694 EX: 3398 ± 694↓	Protein (%) CON: 20.72 ± 4.69 EX: NR ↔
					Fat (%) CON: 20.72 ± 4.69 EX: NR ↔
					CHO (%) CON: 34.22 ± 8.73 EX: NR ↔
Thivel et al. 2013 [74]	n: 10 (4 m, 6 f) Age: 13.2 ± 0.9 y BMI: 33.28 ± 3.65 kg/m²	Modality: Cycling Duration: 3 x 10 min (1.5 min break in between) Intensity: 75% VO2max EE: 243 ± 21 kcal	Pre-Intervention: Standardized breakfast	CON: 1787 ± 404 Bedrest: 1869 ± 294 EX: 1307 ± 304 ↓	Protein (%) CON: 25.45 ± 3.93 EX: 29.75 ± 4.11 ↑
			Post-Intervention: Lunch buffet ad libitum 30 min post-exercise; dinner buffet ad libitum		Fat (%) CON: 14.22 ± 2.24 EX: 16.9 ± 2.34 ↑
					CHO (%) CON: 60.32 ± 6.14 EX: 53.28 ± 6.44 ↓
Thivel et al. 2014 [75]	n: 10 (4 m, 6 f) Age: 13.2 ± 0.9 y BMI: 33.28 ± 3.65 kg/m²	Modality: Cycling Duration: 3 x 10 min (1.5 min break in between) Intensity: 75% VO2max EE: 243 ± 21 kcal	Pre-Intervention: Standardized breakfast	CON: 1787 ± 404 EX: 1306 ± 304 * ↓	Protein (g) CON: 111.21 ± 26.25 EX: 96.18 ± 28.8 ↔
			Post-Intervention: Lunch buffet ad libitum; dinner buffet ad libitum, timing not reported		Fat (g) CON: 24.84 ± 6.29 EX: 21.48 ± 7.31 ↔
				* study arm from Thivel et al. 2013	CHO (g) CON: 276.8 ± 64.48 EX: 180.69 ± 37.19 ↓
	n: 9 (3 m, 6 f) Age: 13.3 ± 0.9 y BMI: 19.11 ± 2.13 kg/m²	Modality: Cycling Duration: 3 x 10 min (1.5 min break in between) Intensity: 75% VO2max EE: NR		CON: 1226 ± 322 EX: 1238 ± 320 ↔	Protein (g) CON: 86.05 ± 25.24 EX: 83.5 ± 25.33 ↔
					Fat (g) CON: 20.06 ± 4.9 EX: 20.05 ± 5.84 ↔
					CHO (g) CON: 174.43 ± 49.04 EX: 180 ± 48.4 ↔
Thivel et al. 2015 [76]	n: 14 m Age: 16.1 ± 0.3 y BMI: 25.8 ± 2.1 kg/m²	Modality: Cycling Duration: 18 ± 3 min Intensity: 75% VO2max EE: 549 ± 3 kcal	Pre-Intervention: Standardized breakfast	CON: 2702 ± 344 EX: 3097 ± 405 ↔	Protein (%) CON: 33.2 ± 3.7 EX: 29.3 ± 6.3 ↔
			Post-Intervention: Lunch buffet ad libitum 30 min post-exercise; snack buffet ad libitum; dinner buffet ad libitum		Fat (%) CON: 13.5 ± 3.8 EX: 19.1 ± 7 ↑
					CHO (%) CON: 52.6 ± 5.6 EX: 51.3 ± 10.5 ↔
		Modality: Rugby session Duration: 60 min Intensity: moderate-to-high EE: 549 ± 3 kcal		CON: 2702 ± 344 EX: 2942 ± 294 ↔	Protein (%) CON: 33.2 ± 3.7 EX: 30.4 ± 4.6 ↔
					Fat (%) CON: 13.5 ± 3.8 EX: 16.6 ± 4.2 ↔
					CHO (%) CON: 52.6 ± 5.6 EX: 51 ± 8.3 ↔
Thivel et al. 2017 [77]	n: 14 (7 m, 7 f) Age: 14.2 ± 1 y BMI: 36.6 ± 5.0 kg/m²	Modality: Cycling Duration: until 25% energy expenditure of energy consumed during lunch on CON day Intensity: 65% VO2max EE: 254 ± 92 kcal	Pre-Intervention: Standardized breakfast	CON: 742 ± 297 EX: 971 ± 225 ↑	Protein (%) CON: 17.3 ± 4.5 EX: 14.9 ± 3.2 ↔
			Post-Intervention: Lunch buffet ad libitum 90 min post-exercise; dinner buffet ad libitum		Fat (%) CON: 21.6 ± 7.8 EX: 36.6 ± 10.9 ↔
					CHO (%) CON: 61.1 ± 10.1 EX: 48.3 ± 9.0 ↔
Thivel et al. 2020 [78]	n: 14 (6 m, 8 f) Age: 12.8 ± 0.9 y BMI: 34.8 ± 5.7 kg/m²	Modality: Cycling Duration: 30 min Intensity: 65% VO2max EE: 177 ± 39 kcal	Pre-Intervention: Standardized breakfast	CON: 1769 ± 532 EX: 1678 ± 501 ↔	Protein (%) CON: NR EX: NR ↔
			Post-Intervention: Lunch buffet ad libitum 105 min post-exercise; dinner buffet ad libitum		Fat (%) CON: NR EX: NR ↔
					CHO (%) CON: NR EX: NR ↔
			Pre-Intervention: Standardized breakfast	CON: 1769 ± 532 EX: 1849 ± 486 ↔	Protein (%) CON: NR EX: NR ↔
			Post-Intervention: Snack to replace exercise induced energy deficit as after-load.		Fat (%) CON: NR EX: NR ↔
			Lunch buffet ad libitum 105 min post-exercise; dinner buffet ad libitum		CHO (%) CON: NR EX: NR ↔
Varley-Campbell et al. 2017 [79]	n: 38 (20 m, 18 f) Age: 13.0 ± 0.3 y BMI: 16.8 ± 2.2 kg/m²	Modality: Cycling Duration: until 1 MJ expended. 31 to 56 min (44 ± 7 min) Intensity: moderate EE: 239 kcal	Pre-Intervention: Same breakfast on all experimental days, standardized snack in SK groups	CON: 1441 ± 113 (SE) CON + SK: 1367 ± 94 (SE) ↔	NR
			Post-Intervention: Lunch pizza meal ad libitum 65 min post- exercise	EX: 1496 ± 111 (SE) EX + SK: 1450 ± 103 (SE) ↔ SK = Snack (containing 239 kcal)	

*CON* Control group, *EX* Exercise group, *VO2max* maximal oxygen uptake, *VT* Ventilation threshold, *EE* Energy expenditure, *NR* Not reported, *SD* standard deviation, *SE* standard error

**↑ **intake significantly higher

**↓ **intake significantly lower

**↔ **no significant change

The studies were published between 2009 and 2020. Most studies were conducted in Europe (*n* = 17; 67%) [60, 62–68, 71–79] followed by Canada and USA (both *n* = 2; 13%) [58, 59, 61, 70]. In general, a frequently implemented design in the included trials was the following: after a preliminary visit where baseline characteristics where collected, participants then visited a controlled environment for one to five intervention days, where data for primary and secondary outcomes where measured.

The studies differed in the number of post-exercise test meals. Thirteen studies examined two post-intervention meals, usually lunch and dinner [61, 63–65, 67, 70–72, 75–78, 80]. Eight studies had one post-intervention meal [58–60, 62, 66, 68, 69, 79]. One study had three test meals [73].

All studies used a cross-over design and compared energy intake in a timeframe of up to 24 h. In all trials, absolute short-term energy intake was the primary or secondary outcome. For the meta-analysis, all 22 trials were eligible.

#### Population characteristics

In total, the 22 trials included 447 participants. The median (Interquartile range, IQR) age was 13.2 (1.1) years, with a range of 9 to 16 years (one trial did not report data on mean age [58]). Twenty-one trials except for one study [58] reported data on sex and 44% of the participants were female. The median (IQR) sample size of the selected studies was 15 (6), the sample size ranged from 9 to 38. Sample size was found to be small in eight studies [64, 65, 68, 69, 71, 73, 74, 78].

Four studies included participants with normal weight [59, 61, 68, 79], 13 studies included participants with overweight or obesity [60, 63–67, 71–74, 76–78], and five studies included both, participants with normal-weight, overweight and/or obesity [58, 62, 69, 70, 75].

#### Exercise characteristics

The 22 trials used eleven different exercise modalities and compared them with control interventions, where participants remained sedentary. Having groups with normal weight and overweight exercising at varying intensities resulted in a total number of 43 exercise conditions, which were compared to a control group in cross-over design. Most of the trials used cycling on an ergometer as the exercise intervention (80%), the second most exercise intervention was walking or running on ground or on a treadmill (12%). Exercise duration ranged from 15 min [59, 67] to 60 min [76], with a median (IQR) duration of 40 (15) minutes. Twenty studies reported the energy expended during the exercise intervention, and two did not [58, 68].

Of the 43 exercise conditions, four (9%) were low in exercise intensity, 26 (60%) used a moderate intensity, and 13 (30%) implemented a high exercise intensity. In most studies (68%), desired exercise intensity was controlled by the previously determined VO2max [60–65, 70–78]. Two studies (9%) determined desired exercise intensity with the use of previously measured ventilation threshold [59, 66] one study determined desired intensity with a percentage of the heart rate reserve [58]. Some studies solely differentiated between high intensity activities (e.g. bouts of 30 s sprints) [68] and low or medium intensity activities (e.g. swimming) [69].

### Summary of study outcomes

#### Total energy intake

At qualitative level, of the exercise conditions compared, three study arms (7%) found a significantly higher energy intake (EI) after exercise [69, 77], ten (23%) found a significant reduction in EI [62, 63, 67, 69, 71–75, 77] and 30 (70%) exercise interventions resulted in no significant change in EI [61–66, 68–70, 73, 75, 76, 78, 79].

**Fig. 2:**
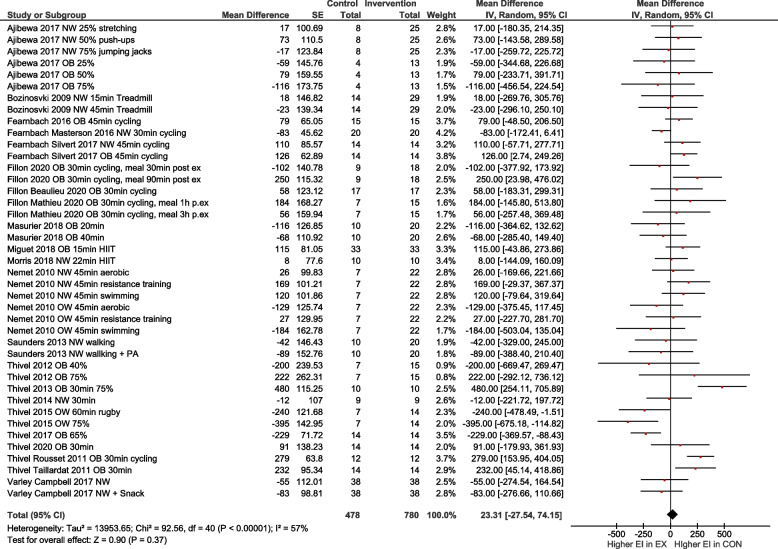
Quantitative analysis for energy intake of randomized controlled trials. In multi-arm trials, the sample size of the shared control group was divided to prevent double counting

In line, the meta-analysis of 41 study arms (exercise *n* = 780 and control *n* = 478) showed no differences in total energy intake between the exercise and control group with a mean difference MD = 23.31 [-27.54, 74.15] kcal (Fig. 2). Sensitivity analyses showed that the results were robust to different correlation coefficients.

**Fig. 3:**
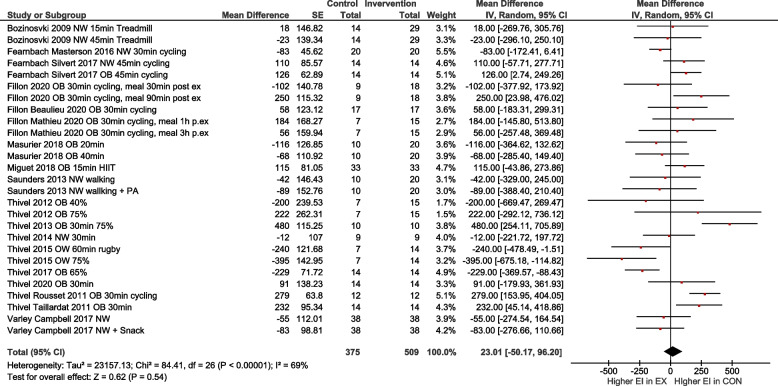
Quantitative analysis for energy intake of randomized controlled trials with low or moderate risk of bias. In multi-arm trials, the sample size of the shared control group was divided to prevent double counting

Overall, the heterogeneity of studies was moderate, with an I^2^ of 57%. After excluding studies with a high risk of bias, I^2^ increased to 69% (Fig. 3).

**Fig. 4:**
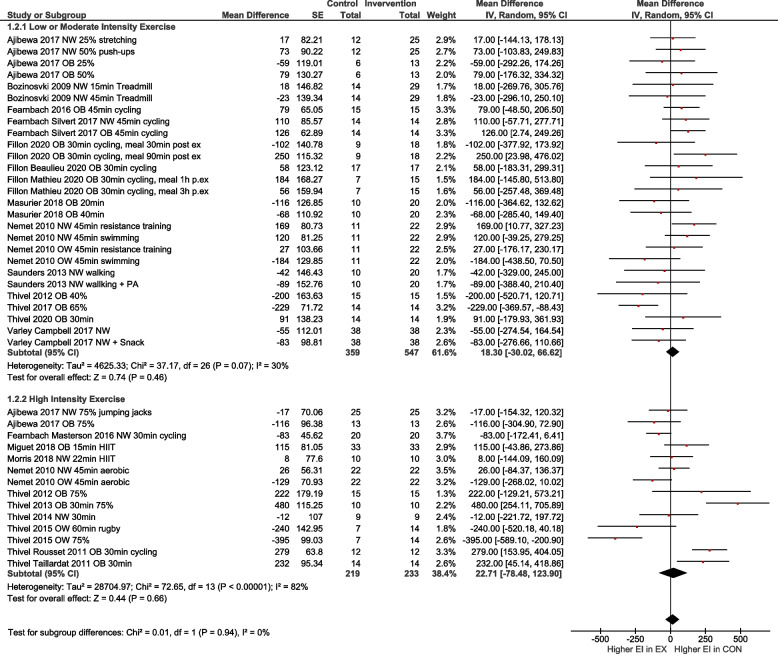
Quantitative analysis for energy intake of randomized controlled trials categorized by low or moderate vs. high intensity exercise. In multi-arm trials, the sample size of the shared control group was divided to prevent double counting

**Fig. 5:**
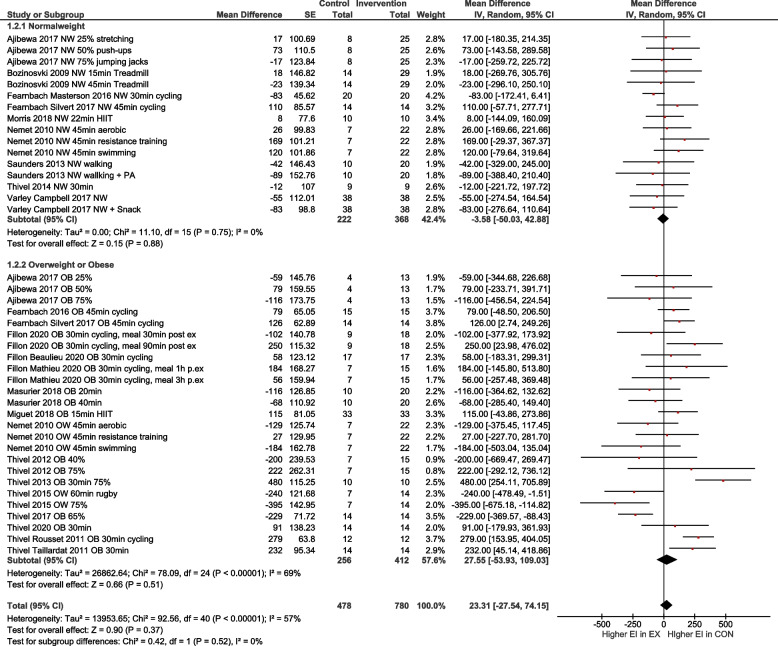
Quantitative analysis for energy intake of randomized controlled trials categorized by subgroups with normal weight vs. overweight or obesity. In multi-arm trials, the sample size of the shared control group was divided to prevent double counting

**Fig. 6:**
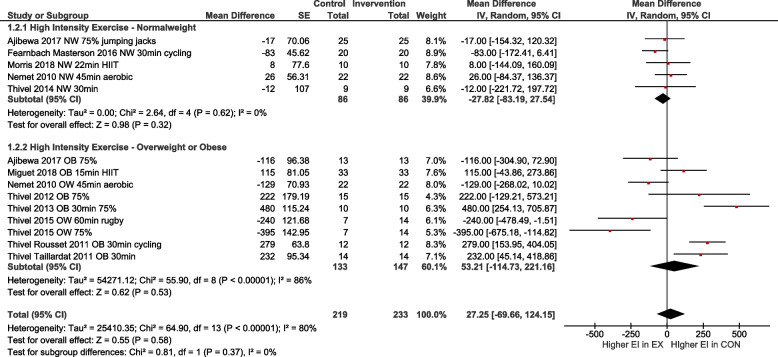
Quantitative analysis for energy intake of randomized controlled trials with high intensity exercise categorized by subgroups with overweight or obesity vs. normal weight. In multi-arm trials, the sample size of the shared control group was divided to prevent double counting

**Fig. 7:**
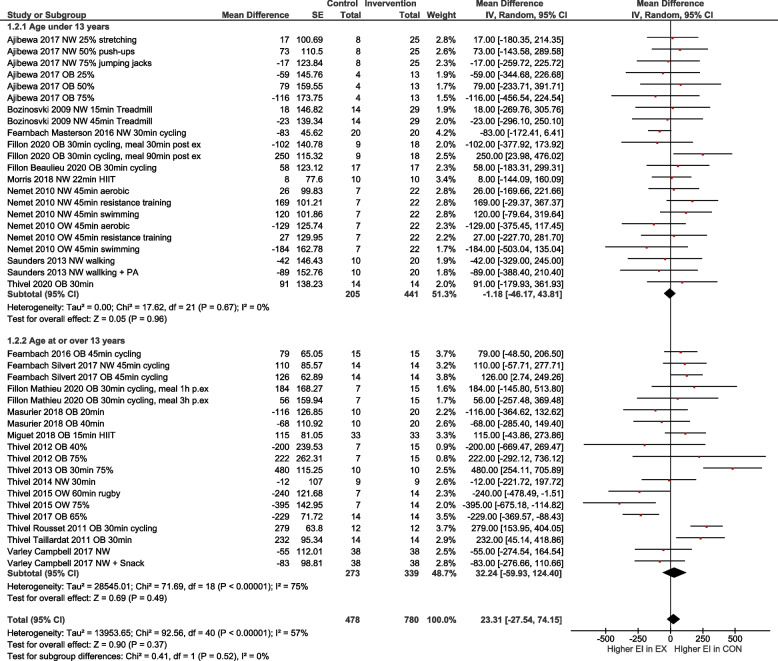
Quantitative analysis for energy intake of randomized controlled trials for subgroups aged < 13 years vs. age ≥ 13 years. In multi-arm trials, the sample size of the shared control group was divided to prevent double counting

To account for differences regarding the exercise condition, age and weight status, subgroup analyses were performed for i) low to moderate exercise vs. high intensity exercise (Fig. 4), ii) normal weight vs. overweight/obese (OW/OB; Fig. 5), iii) high intensity exercise in relation to normal weight vs. OW/OB (Fig. 6) and iv) participants with age < 13 years vs. age ≥ 13 years (Fig. 7). The heterogeneity decreased to I^2^ = 0% when only participants with normal weight were compared, as well as in the group with normal weight exercising at high intensity. No subgroup differences were observed. In particular, there was no influence of intensity of exercise on subsequent energy intake. Sensitivity analyses revealed that the results were robust to different imputed correlation coefficients (corr = 0.3, 0.5, 0.7) (Supporting Information S2).

#### Energy expenditure

Nineteen studies reported the exercise-induced energy expenditure in 33 different exercise conditions, whereas three did not [58, 68, 75]. The energy expended while exercising ranged from 63 ± 7 kcal (in 15 min) [59] to 549 ± 3 kcal (in 60 min) [76], with a median (IQR) energy expenditure of 240 (158) kcal.

#### Macronutrients

**Fig. 8:**
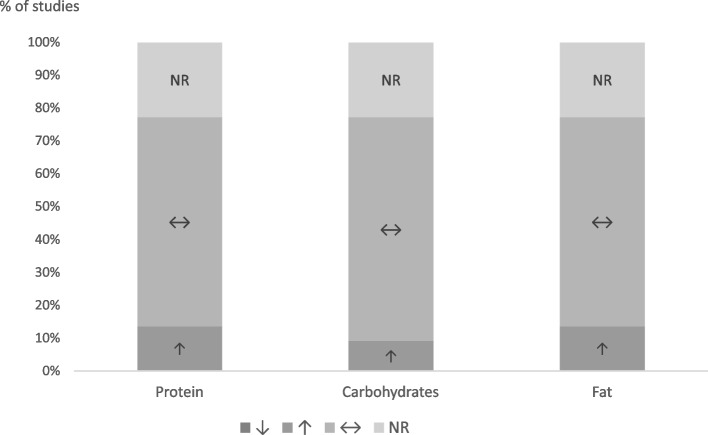
Changes in macronutrient intake (protein, carbohydrates, fat) after exercise intervention compared to sedentary control. ↑: intake was higher after exercise; ↓: intake was lower after exercise; ↔ : no significant differences; NR: not reported

Seventeen studies investigated macronutrient intake (Fig. 8), while five did not [58, 59, 69, 72, 79]. Of them, fifteen studies reported data on macronutrient intake either in grams or percentage of total food intake, and two studies solely stated whether macronutrient intake differed between intervention groups [64, 78]. Regarding protein, three studies found a significant increase in protein-intake after exercise [61, 65, 74], whereas the remaining 14 reported no significant changes. With regard to fat, three studies observed a significant increase in fat-intake after exercise [61, 74, 76], the remaining 14 found no such relationship. Finally, two studies reported a decrease in carbohydrate intake after exercise [74, 75] which was not the case in the other 15 studies. Overall, physical activity had no substantial effect on macronutrient intake.

#### Appetite Sensations

**Fig. 9:**
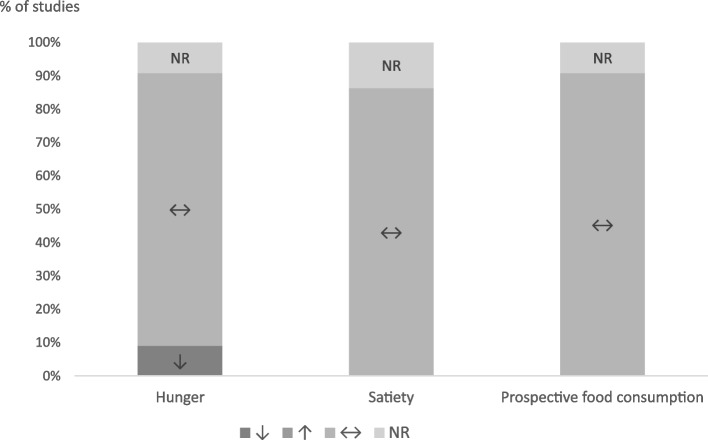
Changes in hunger, satiety, prospective food consumption after exercise intervention compared to sedentary control. ↑: higher after exercise; ↓: lower after exercise; ↔ : no significant differences; NR: not reported

All but three studies [61, 69, 71] examined hunger, satiety and prospective food consumption, one did report only hunger and prospective food consumption [71] (Fig. 9). Six studies also investigated the desire to eat [59, 63–65, 67, 78] and two studies used the Leeds food preference score [64, 65]. Summarized across studies, there was no significant effect on hunger, satiety, and prospective food consumption, with the exception of two studies: Bozinovski et al. [59] found hunger significantly attenuated after short duration exercise compared to long duration exercise and control and Fillon et al. [63] reported significantly reduced hunger in both exercise groups compared to control. Overall, physical activity had no substantial effect on appetite sensations.

### Risk of bias

The risk of bias assessment is presented in Table 2. The overall risk of bias was low in seven studies (31%), with some concerns in eleven studies (50%) and high in four studies (18%). One of the major methodological problems of the studies was that the data used to determine the outcome were not analysed according to a pre-specified analysis plan (which was completed before the outcome data were available for analysis), as required in domain 5 (D5) [57]. Another common issue relates to the duration that elapsed between interventions. Due to the within-subject design of the trials, studies were only classified as low-risk if no more than 28 days elapsed between interventions to rule out a significant alteration in metabolism during this time. However, several trials did not report any time between interventions, so they were classified as “some-concerns”, as required in domain 4 (D4) [57]. Most studies did not do a power calculation, two reported to be underpowered [59, 61].

**Table 2:**
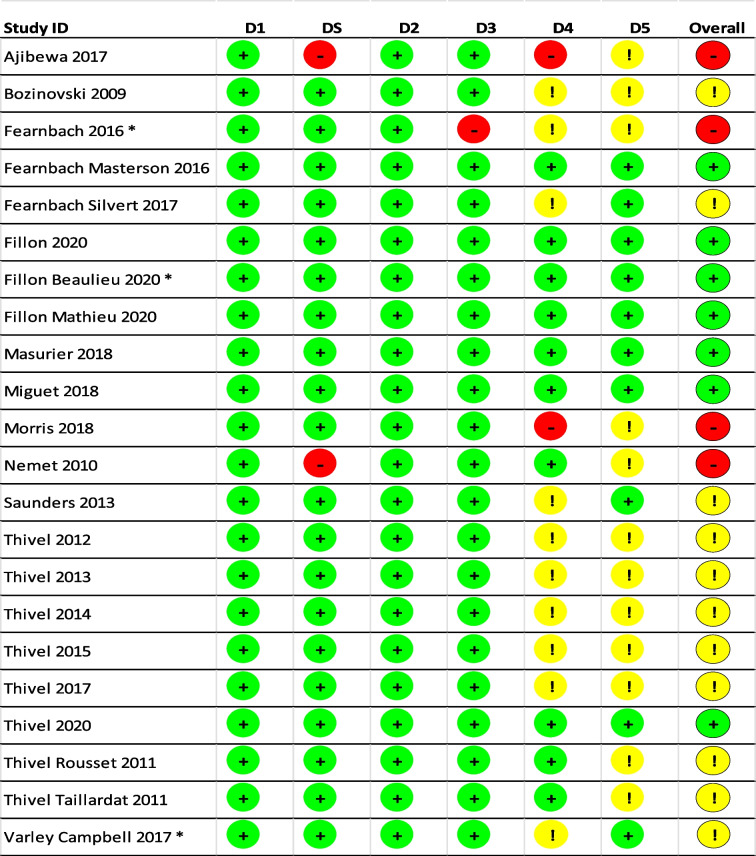
2 Risk of bias. +: Low risk, !: Some concerns, -: High risk, D1: Randomization process, D2: Deviations from the intended interventions, D3: Missing outcome data, D4: Measurement of the outcome, D5: Selection of the reported result. All studies analyzed as intention-to-treat except: * = per-protocol analysis

## Discussion

The aim of this study was to analyze the effects of acute exercise on energy intake under controlled conditions in children and adolescents to contribute to our understanding of energy balance regulation. The first research question analyzed whether acute exercising is followed by increased food intake. We found that children and adolescents did not alter their caloric and macronutrient intake after exercise tasks compared to their sedentary controls. These findings are in line with the previous study conducted in children and adolescents by Thivel et al. [44]. They are also largely consistent with meta-analyses conducted in adults on the acute (24 h) and short-term (up to 14 days) impact of exercise on food intake [37, 38].

The second question addressed whether training intensity, duration, and type of exercise affected subsequent energy intake, and the third question examined the influence of body weight status on the findings. Our data are partly in line with those of the earlier study by Thivel et al. [44]. In accordance with their findings, the group with normal weight did not alter their energy intake following exercise. However, they found a reducing effect of high-intensity exercise on energy intake only in the subgroup with obesity. The authors concluded that performing exercise at high intensity may have a transient anorexigenic effect in adolescents with obesity. These findings could not be confirmed in the present meta-analysis. This is most likely because stricter inclusion criteria were applied, and new high-quality randomized controlled trials were included in the analysis.

As shown in this meta-analysis, children and adolescents did not acutely compensate for an exercise intervention with increased food intake, regardless of age group, body weight status or exercise intensity. This results, on average, in a short-term negative energy balance, assuming an otherwise neutral energy balance. In the trials examined, a median of 240 kcal was expended during exercise. This corresponds to more than 10% of the daily caloric needs of a moderately active 10- to 13-year-old child [81]. A long-term caloric deficit of this magnitude resulting from exercise would lead to loss of adipose tissue or, if compensated for, contribute to a stable bodyweight, improved body composition and aid in healthy weight management [82–84]. This weight-reducing effect indicated by the results reported here was also demonstrated for adults with overweight in an umbrella review [85]. Since the actual weight loss is often less than expected, compensatory mechanisms such as increased sedentary behavior or a reduction in metabolic activity to keep energy balance stable have been suggested [86, 87]. This assumption is referred to as the “constrained total energy expenditure model” [88], but its validity is the subject of current debate [89]. Data on whether and under what circumstances exercise leads to a subsequent reduction in non-exercise physical activity are mixed [30, 32, 86, 90, 91].

Data on food intake in relation to longer periods of physical activity training are based on self-reported data. Thus, results from such longer studies cannot be directly compared with short-term, highly controlled conditions such as those reviewed here. However, the effect of at least 10 weeks of regular physical activity was systematically reviewed by Schwartz et al. [92]. Adolescents with obesity were found to reduce their self-reported food intake in response to several weeks of sports intervention. Similarly, the longer-term effects of an exercise intervention on energy intake have been studied in adults. In their meta-analysis, Beaulieu et al. [36] investigated the impact of exercise training interventions (median duration 12 weeks) on energy intake and appetite in adults with overweight/obesity. They found that no significant changes in food intake or appetite over the course of an exercise intervention occurred. In summary, the above results all point in the direction that, on average, exercise does not lead to an increase in food intake.

We demonstrated that not only caloric intake, but also macronutrient consumption remained unaffected by the intervention. This is in line with the findings by Thivel et al. [44] and is still the case when long-term interventions (at least 10 weeks) are examined under less controlled conditions, as reported by Schwartz et al. [92]. Similarly, adults also do not alter their macronutrient intake in response to exercise as presented by Donnelly et al. [37] and Beaulieu et al. [36]. Contrary to popular belief, this meta-analysis showed no increase of appetite after acute exercise. This is in accordance with the literature on adults [36, 37], and youth alike [44]. The influence of exercise on appetite-related hormones in children and adolescents has been sparsely studied to date [93]. In contrast to most studies in adults, Rumbold et al. [94] found increased levels of the hunger-inducing hormone Ghrelin after acute exercise in adolescent females. Consistent with the findings in adults, Prado et al. [95] demonstrated a significant increase in the hunger-reducing hormone Peptide Y in adolescent girls with obesity following 30 min of exercise.

This study has strengths and limitations. Strengths include adherence to PRISMA guidelines and exclusive examination of randomized controlled trials. The risk of bias in the studies was assessed using the Cochrane RoB-2-tool. Additionally, studies utilizing self-reported dietary intake, food frequency questionnaires, and similar potentially biased data acquisition methods were excluded [96–98]. All studies employed a crossover design, where subjects acted as their own controls, ensuring high comparability. The heterogeneous study population, comprising both sexes, all levels of fitness, and a broad body weight range, was chosen to closely reflect real-life conditions, enhancing generalizability. However, there are limitations. Sample sizes were often small, and studies predominantly focused on older children and adolescents. Moreover, the short-term duration of the studies prevents determination of whether compensatory food intake occurred in subsequent hours (e.g., at night or the next morning). When studies did not provide sufficient data from a paired analysis, a correlation coefficient of 0.5 was applied. Sensitivity analysis revealed stable results when using 0.3 and 0.7 as correlation coefficient. Nevertheless, a degree of uncertainty for data interpretation remains. Additionally, the applicability of results from randomized controlled trials in laboratory settings to the everyday lives of children and adolescents remains uncertain. Environmental factors could lead to overconsumption of palatable, high-energy-dense foods and large portions [6], even after exercising. In many study designs, accurately determining whether caloric intake met or exceeded energy needs was challenging. Solely Thivel et al. [43] utilized a calorimetric chamber, to precisely measure energy balance. Only two of the included studies accounted for fluid intake through beverages, standardizing post-intervention intake across participants [59, 79]. It is conceivable that increased drinking, due to thirst after exercise, led to a feeling of fullness and reduced food intake, as distension of the gastric wall is a key signal generator for satiety [99–101].

## Conclusions

Engaging in physical activity has no significant effect on the subsequent energy or macronutrient intake of children and adolescents, nor on their appetite sensations, compared with a sedentary control group within a single day. However, exercise acutely raises energy expenditure and thus may help to control energy balance. The findings of this meta-analysis and other reviews related to this field support the importance of physical activity in promoting weight loss and improving body composition along with a balanced diet, as children and adolescents are not expected to eat more as a result of exercise. Additionally, there was no increase in appetite, which would be detrimental to weight reduction efforts. These findings were derived from controlled conditions. Considering other literature from the field, environmental factors could lead to overconsumption of palatable, high-energy-dense foods and large portions and/or increased sedentary behavior after exercising, compensating for the increased energy expenditure. Therefore, monitoring and reflecting on individual behavior after exercising appears to be useful for individual recommendations and countermeasures.

## Supplementary Information

Supplementary Material 1.

Supplementary Material 2.

## Data Availability

The datasets used and analyzed during the current study are available from the corresponding author on reasonable request.

## Abbreviations

IQR: Interquartile range
kcal: Kilocalories
MD: Mean difference
SE: Standard error
BMI: Body mass index
RMR: Resting metabolic rate
PRISMA: Preferred Reporting Items for Systematic Reviews and Meta-Analyses
PICOS: Participants, interventions, comparators, outcome, study design
VO2max: Maximum oxygen consumption
HRmax: Maximum heart rate
SD: Standard deviation
CI: Confidence interval
RoB 2: Risk-of-Bias tool for randomized crossover trials
EI: Energy intake
OW: Overweight
OB: Obese
