# Publisher Correction: Impact of physical activity on caloric and macronutrient intake in children and adolescents: a systematic review and meta-analysis of randomized controlled trials

**DOI:** 10.1186/s12966-024-01651-1

**Published:** 2024-09-23

**Authors:** Heiko Hahn, Manuel Friedel, Claudia Niessner, Stephan Zipfel, Isabelle Mack

**Affiliations:** 1 Department of Psychosomatic Medicine and Psychotherapy, University Medical Hospital Tübingen, Osianderstr. 5, Tübingen, 72076 Germany; 2 Institute of Sports and Sport Science, Karlsruhe Institute of Technology, Engler-Bunte-Ring 15, Karlsruhe, 76131 Germany

**Correction** **:** **Int J Behav Nutr Phys Act 21, 76 (2024)**

**https://doi.org/10.1186/s12966-024-01620-8**

After the publication of the Original Article [1], a typesetting error was identified in Table 1. Visible lines in the XML version and incorrect spacings in the PDF version caused data from the same category to appear separated.

The correct format of Table 1 is as follows:

**Table 1:** Overview of characteristics of trials

Study	Population	Exercise characteristics	Meal characteristics	Absolute Energy Intake in kcal (mean ± SD) Statistically significant change in intake (↑, ↓, ↔)	Macronutrients Statistically significant change in Intake (↑, ↓, ↔)
Ajibewa et al. 2017	n: 26 (NR) Age: 7 – 11 y BMI: normal weight, not further specified	Modality: Static stretching, standing and yoga poses Duration: 20 × 2 min Intensity: Resting heart rate + heart rate reserve * 25% EE: NR	Pre-Intervention: Three standardized meals Post-Intervention: One meal ad libitum	CON: 999 ± 62 (SE) EX: 982 ± 50 (SE) ↔	NR
		Modality: Push-ups, sit-ups, and age-appropriate calisthenics Duration: 20 × 2 min Intensity: Resting heart rate + heart rate reserve * 50% EE: NR		CON: 999 ± 62 (SE) EX: 926 ± 63 (SE) ↔	
		Modality: Vigorous calisthenics (e.g., jumping jacks) Duration: 20 × 2 min Intensity: Resting heart rate + heart rate reserve * 75% EE: NR		CON: 999 ± 62 (SE) EX: 1016 ± 76 (SE) ↔	
	n: 13 (NR) Age: 7 – 11 y BMI: obese, not further specified	Modality: Static stretching, standing and yoga poses Duration: 20 × 2 min Intensity: Resting heart rate + heart rate reserve * 25% EE: NR		CON: 1145 ± 88 (SE) EX: 1204 ± 71 (SE) ↔	
		Modality: Push-ups, sit-ups, and age-appropriate calisthenics Duration: 20 × 2 min Intensity: Resting heart rate + heart rate reserve * 50% EE: NR		CON: 1145 ± 88 (SE) EX: 1066 ± 89 (SE) ↔	
		Modality: Vigorous calisthenics (e.g., jumping jacks) Duration: 20 × 2 min Intensity: Resting heart rate + heart rate reserve * 75% EE: NR		CON: 1145 ± 88 (SE) EX: 1261 ± 103 (SE) ↔	
Bozinovski et al. 2009	n: 29 (14 m, 15 f) Age: 12.1 ± 0.4 y BMI: 54.3rd ± 5.3 percentile	Modality: Treadmill Duration: 15 min Intensity: Ventilation Threshold EE: 63 ± 7 kcal	Pre-Intervention: Standardized breakfast Post-intervention: 250 mL water, pizza meal ad libitum 30 min post-exercise	CON: 1078 ± 101 (SE) EX: 1060 ± 103 (SE) ↔	NR
		Modality: Treadmill Duration: 45 min Intensity: Ventilation Threshold EE: 189 ± 14 kcal		CON: 1078 ± 101 (SE) EX: 1101 ± 92 (SE) ↔	
Fearnbach et al. 2016	n: 15 m Age: 13.8 ± 1.5 y BMI: 31.8 ± 4.2 kg/m^2^	Modality: Cycling Duration: 45 min Intensity: 65% VO2max EE: 399 ± 75 kcal	Pre-Intervention: Standardized breakfast Post-Intervention: Buffet meal ad libitum 30 min post-exercise	CON: 1116 ± 243 EX: 1037 ± 260 ↓	Protein (%) CON: 29.4 ± 7.2 EX: 30.5 ± 6.7 ↔ Fat (%) CON: 16.5 ± 4.2 EX: 16.6 ± 4.2 ↔ CHO (%) CON: 53.4 ± 11.0 EX: 52.3 ± 10.5 ↔
Fearnbach, Masterson et al. 2016	n: 20 (12 m, 8 f) Age: 10.3 ± 1.1 y BMI: 41.6 ± 21.7 percentile	Modality: Cycling Duration: 30 min Intensity: 70% VO2max EE: 534 ± 263 kcal	Pre-Intervention: Standardized breakfast Post-Intervention: Standardized snack; lunch and dinner meal ad libitum, timing not reported	CON: 2088 ± 497 EX: 2171 ± 566 ↔	Protein (kcal) CON: 196 ± 67 EX: 207 ± 68 ↑ Fat (kcal) CON: 655 ± 155 EX: 694 ± 181 ↑ CHO (kcal) CON: 1235 ± 295 EX: 1269 ± 337 ↔
Fearnbach, Silvert et al. 2017	n: 14 m Age: 13.9 ± 1.1 y BMI: 31.6 ± 4.5 kg/m^2^	Modality: Cycling Duration: 45 min Intensity: 65% VO2max EE: 373 ± 57 kcal	Pre-Intervention: Standardized breakfast Post-Intervention: Buffet meal ad libitum 30 min post-exercise	CON: 1091 ± 252 EX: 965 ± 214 ↓	Protein (%) CON: 31.1 ± 5.5 EX: 31.8 ± 4.8 ↔ Fat (%) CON: 17.5 ± 3.2 EX: 17.1 ± 3.3 ↔ CHO (%) CON: 50.8 ± 8.3 EX: 50.4 ± 7.7 ↔
	n: 14 m Age: 13.7 ± 1.1 y BMI: 19.2 ± 3.2 kg/m^2^	Modality: Cycling Duration: 45 min Intensity: 65% VO2max EE: 241 ± 93 kcal		CON: 854 ± 362 EX: 744 ± 246 ↔	Protein (%) CON:28.0 ± 7.2 EX: 28.1 ± 5.3 ↔ Fat (%) CON: 16.4 ± 4.9 EX: 16.1 ± 4 ↔ CHO (%) CON: 55.1 ± 11.1 EX: 52.2 ± 8.6 ↔
Fillon et al. 2020	n: 18 (12 m, 6 f) Age: 12.7 ± 1.3 y BMI: 33.3 ± 6.5 kg/m^2^	Modality: Cycling Duration: 30 min Intensity: 65% VO2max EE: 169 ± 44 kcal	Pre-Intervention: Standardized breakfast Post-Intervention: Buffet lunch meal ad libitum 30 min post-exercise; dinner buffet meal ad libitum	CON: 2175 ± 330 EX: 2277 ± 476 ↔	Protein (%) CON: 21.3 ± 2.5 EX: 21.0 ± 2.0 ↔ Fat (%) CON: 30.7 ± 5.8 EX: 31.2 ± 4.8 ↔ CHO (%) CON: 47.8 ± 7.4 EX: 47.4 ± 6.1 ↔
			Pre-Intervention: Standardized breakfast Post-Intervention:	CON: 2175 ± 330 EX: 1925 ± 360 ↓	Protein (%) CON: 21.3 ± 2.5 EX: 20.6 ± 2.3 ↔
			Buffet lunch meal ad libitum 90 min post- exercise; dinner buffet meal ad libitum		Fat (%) CON: 30.7 ± 5.8 EX: 30.5 ± 5.7 ↔ CHO (%) CON: 47.8 ± 7.4 EX: 48.7 ± 7.3 ↔
Fillon, Beaulieu et al. 2020	n: 17 (9 m, 8 f) Age: 12.8 ± 1.4 y BMI: 33.4 ± 5.7 kg/m^2^	Modality: Cycling Duration: 30 min Intensity: 65% VO2max EE: 135 kcal ± NR	Pre-Intervention: Not clearly reported Post-Intervention: Lunch ad libitum immediately post- exercise; dinner buffet ad libitum	CON: 1997 ± 514 EX: 1939 ± 501 ↔	Protein (%) CON: NR EX: NR ↔ Fat (%) CON: NR EX: NR ↔ CHO (%) CON: NR EX: NR ↔
Fillon, Mathieu et al. 2020	n: 15 (6 m, 9 f) Age: 13.1 ± 1.4 y BMI: 34.7 ± 6.0 kg/m^2^	Modality: Cycling Duration: 30 min Intensity: 65% VO2max EE: 186 ± 52 kcal	Pre-Intervention: Standardized breakfast Post-Intervention: Lunch meal ad libitum 180 min post-exercise; dinner buffet meal ad libitum	CON: 2004 ± 430 EX: 1948 ± 416 ↔	Protein (%) CON: 22.0 ± 2.5 EX: 24.1 ± 3.7 ↔ Fat (%) CON: 30.8 ± 4.8 EX: 27.1 ± 7.0 ↔ CHO (%) CON: 46.9 ± 6.4 EX: 48.7 ± 8.9 ↔
			Pre-Intervention: Standardized breakfast Post-Intervention: Lunch meal ad libitum 60 min post-exercise; dinner buffet meal ad libitum	CON: 2004 ± 430 EX: 1820 ± 459 ↔	Protein (%) CON: 22.0 ± 2.5 EX: 23.5 ± 3.7 ↑ Fat (%) CON: 30.8 ± 4.8 EX: 26.7 ± 8.1 ↔ CHO (%) CON: 46.9 ± 6.4 EX: 49.0 ± 10.5 ↔
Masurier et al. 2018	n: 20 f Age: 13.3 ± 1.0 y BMI: 31.6 ± 3.9 kg/m^2^	Modality: cycling Duration: 20 min Intensity: Ventilation Threshold (54.1 ± 5.4% of VO2max) EE: 117 ± 22 kcal	Pre-Intervention: Standardized breakfast Post-Intervention: Buffet meal ad libitum 30 min post-exercise	CON: 738 ± 320 EX: 854 ± 450 ↔	Protein (%) CON: 16.3 ± 4.2 EX: 18.2 ± 4.5 ↔ Fat (%) CON: 9.7 ± 2.6 EX: 11.5 ± 11.5 ↔ CHO (%) CON: 71.1 ± 13.1 EX: 69.9 ± 7.4 ↔
		Modality: Cycling Duration: 40 min Intensity: Ventilation Threshold (54.1 ± 5.4% of VO2max) EE: 235 ± 44 kcal		CON: 738 ± 320 EX: 806 ± 375 ↔	Protein (%) CON: 16.3 ± 4.2 EX: 17.5 ± 3.2 ↔ Fat (%) CON: 9.7 ± 2.6 EX: 11.1 ± 2.7 ↔ CHO (%) CON: 71.1 ± 13.1 EX: 71.0 ± 5.4 ↔
Miguet et al. 2018	n: 33 (12 m, 21 f) Age: 13.0 ± 0.9 y BMI: 35 ± 4.3 kg/m^2^	Modality: Cycling Duration: 15 min (5 × 2 min high, 30 s. Low intensity) Intensity: High intensity intervals EE: 102 ± 21 kcal	Pre-Intervention: Standardized breakfast Post-Intervention: Lunch buffet ad libitum 30 min post-exercise; dinner buffet ad libitum	CON: 2177 ± 471 EX: 2062 ± 460 ↓	Protein (%) CON: 22.6 ± 3.4 EX: 22.7 ± 3.3 ↔ Fat (%) CON: 32.7 ± 6.1 EX: 31.9 ± 5.9 ↔ CHO (%) CON: 45.3 ± 7.1 EX: 46.1 ± 7.03 ↔
Morris et al. 2018	n: 10 (5 m, 5 f) Age: 9.8 ± 0.6 y BMI: 18.3 ± 2.6 kg/m^2^	Modality: Sprints Duration: 22 min (8 × 30 s) Intensity: high intensity intervals EE: NR	Pre-Intervention: Same breakfast on both experimental days Post-Intervention: Lunch meal ad libitum 5–10 min post-exercise	CON: 500 ± 69 EX: 492 ± 84 ↔	Protein (g) CON: 12.7 ± 1.2 EX: 12.5 ± 1.5 ↔ Fat (g) CON: 23.4 ± 3.2 EX: 22.9 ± 3.7 ↔ CHO (g) CON: 60.2 ± 9.5 EX: 58.4 ± 11.6 ↔
Nemet et al. 2010	n: 22 (7 m, 15 f) Age: 9.1 ± 0.6 y BMI: 23.9 ± 0.6 kg/m^2^	Modality: Aerobic games Duration: 45 min Intensity: high EE: 9.6 kcal / kg Bodyweight (BW)	Pre-Intervention: Controlled diet 24 h prior to experimental days Post-Intervention: Lunch buffet ad libitum 30–45 min post-exercise	CON: 806 ± 51 (SE) EX: 935 ± 81 (SE) ↑	NR
		Modality: Swimming Duration: 45 min Intensity: moderate EE: 7.6 kcal / kg BW		CON: 806 ± 51 (SE) EX: 990 ± 106 (SE) ↑	
		Modality: Resistance Duration: 45 min Intensity: moderate EE: 6.3 kcal / kg BW		CON: 806 ± 51 (SE) EX: 779 ± 84 (SE) ↔	
	n: 22 (5 m, 17 f) Age: 9.4 ± 0.3 y BMI: 17.0 ± 0.4 kg/m^2^	Modality: Aerobic games Duration: 45 min Intensity: high EE: 10.2 kcal / kg BW		CON: 604.7 ± 64.5 (SE) EX: 579.3 ± 34.1 (SE) ↔	
		Modality: Swimming Duration: 45 min Intensity: moderate EE: 8.1 kcal / kg BW		CON: 604.7 ± 64.5 (SE) EX: 484.9 ± 44.4 (SE) ↔	
		Modality: Resistance Duration: 45 min Intensity: moderate EE: 6.9 kcal / kg BW		CON: 604.7 ± 64.5 (SE) EX: 435.9 ± 41.8 (SE) ↓	
Saunders et al. 2013	n: 20 (8 m, 12 f) Age: 12.2 ± 0.9 y BMI: 18.6 ± 4.3 kg/m^2^	Modality: Walking Duration: 2 min every 20 min (42 min total) Intensity: low EE: 744 ± 141 kcal (in 9 h)	Pre-Intervention: Standardized breakfast Post-Intervention: Standardized lunch; dinner buffet ad libitum 3 h post-exercise	CON: 1176 ± 459 EX: 1218 ± 467 ↔	Protein (%) CON: 10.68 ± 2.51 EX: 11.46 ± 3.32 ↔ Fat (%) CON: 34.51 ± 7.3 EX: 33.3 ± 8,1 ↔ CHO (%) CON: 54.81 ± 7.6 EX: 55.24 ± 9 ↔
		Modality: Walking + Treadmill Duration: 2 min every 20 min (42 min total) + 40 min treadmill Intensity: 20 min at 60% VO2max + 20 min at 30% VO2max EE: 970 ± 219 kcal (in 9 h)		CON: 1176 ± 459 EX: 1265 ± 503 ↔	Protein (%) CON: 10.68 ± 2.51 EX: 10.71 ± 3.13 ↔ Fat (%) CON: 34.51 ± 7.3 EX: 35.61 ± 9.38 ↔ CHO (%) CON: 54.81 ± 7.6 EX: 53.7 ± 9.1 ↔
Thivel, Isacco, Rousset et al. 2011	n: 12 (5 m, 7 f) Age: 14.4 ± 1.5 y BMI: 35.1 ± 7.6 kg/m^2^	Modality: Cycling Duration: 30 min Intensity: 70% VO2max EE: 298 ± 28 kcal	Pre-Intervention: Standardized breakfast Post-Intervention: Lunch buffet ad libitum 30 min post-exercise; dinner buffet ad libitum	CON: 2214 ± 222 EX: 1935 ± 220 ↓	Protein (kcal) CON: 192 ± 33 EX: 206 ± 42 NR Fat (kcal) CON: 327 ± 66 EX: 373 ± 64 NR CHO (kcal) CON: 453 ± 120 EX: 367 ± 76 NR
Thivel, Isacco, Taillardat et al. 2011	n: 14 (7 m, 7 f) Age: 14.1 ± 1.8 y BMI: 33.9 ± 7.5 kg/m^2^	Modality: Cycling Duration: 3 × 10 min (2 min rest in between) Intensity: 70% VO2max EE: 299 ± 29 kcal	Pre-Intervention: Standardized breakfast Post-Intervention: Lunch buffet ad libitum 30 min post-exercise; dinner buffet ad libitum	CON: 1808 ± 301 EX: 1576 ± 394 ↓	NR
Thivel et al. 2012	n: 15 m Age: 13.5 ± 0.9 y BMI: 30.7 ± 4.1 kg/m^2^	Modality: Cycling Duration: 59 ± 6 min Intensity: 40% VO2max EE: 336 ± 50 kcal	Pre-Intervention: Calibrated breakfast Post-Intervention: Lunch buffet ad libitum 30 min post-exercise; dinner buffet ad libitum; breakfast buffet ad libitum the next morning	CON: 3620 ± 694 EX: 3820 ± 584 ↔	Protein (%) CON: 20.72 ± 4.69 EX: 19.5 ± 3.21 ↔ Fat (%) CON: 20.72 ± 4.69 EX: 43.44 ± 9.58 ↔ CHO (%) CON: 34.22 ± 8.73 EX: 37.04 ± 10.36 ↔
		Modality: Cycling Duration: 30 ± 3 min Intensity: 75% VO2max EE: 332 ± 47 kcal		CON: 3620 ± 694 EX: 3398 ± 694 ↓	Protein (%) CON: 20.72 ± 4.69 EX: NR ↔ Fat (%) CON: 20.72 ± 4.69 EX: NR ↔ CHO (%) CON: 34.22 ± 8.73 EX: NR ↔
Thivel et al. 2013	n: 10 (4 m, 6 f) Age: 13.2 ± 0.9 y BMI: 33.28 ± 3.65 kg/m^2^	Modality: Cycling Duration: 3 × 10 min (1.5 min break in between) Intensity: 75% VO2max EE: 243 ± 21 kcal	Pre-Intervention: Standardized breakfast Post-Intervention: Lunch buffet ad libitum 30 min post-exercise; dinner buffet ad libitum	CON: 1787 ± 404 Bedrest: 1869 ± 294 EX: 1307 ± 304 ↓	Protein (%) CON: 25.45 ± 3.93 EX: 29.75 ± 4.11 ↑ Fat (%) CON: 14.22 ± 2.24 EX: 16.9 ± 2.34 ↑ CHO (%) CON: 60.32 ± 6.14 EX: 53.28 ± 6.44 ↓
Thivel et al. 2014	n: 10 (4 m, 6 f) Age: 13.2 ± 0.9 y BMI: 33.28 ± 3.65 kg/m^2^	Modality: Cycling Duration: 3 × 10 min (1.5 min break in between) Intensity: 75% VO2max EE: 243 ± 21 kcal	Pre-Intervention: Standardized breakfast Post-Intervention: Lunch buffet ad libitum; dinner buffet ad libitum, timing not reported	CON: 1787 ± 404 EX: 1306 ± 304 * ↓	Protein (g) CON: 111.21 ± 26.25 EX: 96.18 ± 28.8 ↔ Fat (g) CON: 24.84 ± 6.29 EX: 21.48 ± 7.31 ↔ CHO (g) CON: 276.8 ± 64.48 EX: 180.69 ± 37.19 ↓
				* Study arm from Thivel et al. 2013	
	n: 9 (3 m, 6 f) Age: 13.3 ± 0.9 y BMI: 19.11 ± 2.13 kg/m^2^	Modality: Cycling Duration: 3 × 10 min (1.5 min break in between) Intensity: 75% VO2max EE: NR		CON: 1226 ± 322 EX: 1238 ± 320 ↔	Protein (g) CON: 86.05 ± 25.24 EX: 83.5 ± 25.33 ↔ Fat (g) CON: 20.06 ± 4.9 EX: 20.05 ± 5.84 ↔ CHO (g) CON: 174.43 ± 49.04 EX: 180 ± 48.4 ↔
Thivel et al. 2015	n: 14 m Age: 16.1 ± 0.3 y BMI: 25.8 ± 2.1 kg/m^2^	Modality: Cycling Duration: 18 ± 3 min Intensity: 75% VO2max EE: 549 ± 3 kcal	Pre-Intervention: Standardized breakfast Post-Intervention: Lunch buffet ad libitum 30 min post-exercise; snack buffet ad libitum; dinner buffet ad libitum	CON: 2702 ± 344 EX: 3097 ± 405 ↔	Protein (%) CON: 33.2 ± 3.7 EX: 29.3 ± 6.3 ↔ Fat (%) CON: 13.5 ± 3.8 EX: 19.1 ± 7 ↑
					CHO (%) CON: 52.6 ± 5.6 EX: 51.3 ± 10.5 ↔
		Modality: Rugby session Duration: 60 min Intensity: moderate-to-high EE: 549 ± 3 kcal		CON: 2702 ± 344 EX: 2942 ± 294 ↔	Protein (%) CON: 33.2 ± 3.7 EX: 30.4 ± 4.6 ↔ Fat (%) CON: 13.5 ± 3.8 EX: 16.6 ± 4.2 ↔ CHO (%) CON: 52.6 ± 5.6 EX: 51 ± 8.3 ↔
Thivel et al. 2017	n: 14 (7 m, 7 f) Age: 14.2 ± 1 y BMI: 36.6 ± 5.0 kg/m^2^	Modality: Cycling Duration: until 25% energy expenditure of energy consumed during lunch on CON day Intensity: 65% VO2max EE: 254 ± 92 kcal	Pre-Intervention: Standardized breakfast Post-Intervention: Lunch buffet ad libitum 90 min post-exercise; dinner buffet ad libitum	CON: 742 ± 297 EX: 971 ± 225 ↑	Protein (%) CON: 17.3 ± 4.5 EX: 14.9 ± 3.2 ↔ Fat (%) CON: 21.6 ± 7.8 EX: 36.6 ± 10.9 ↔ CHO (%) CON: 61.1 ± 10.1 EX: 48.3 ± 9.0 ↔
Thivel et al. 2020	n: 14 (6 m, 8 f) Age: 12.8 ± 0.9 y BMI: 34.8 ± 5.7 kg/m^2^	Modality: Cycling Duration: 30 min Intensity: 65% VO2max EE: 177 ± 39 kcal	Pre-Intervention: Standardized breakfast Post-Intervention: Lunch buffet ad libitum 105 min post-exercise; dinner buffet ad libitum	CON: 1769 ± 532 EX: 1678 ± 501 ↔	Protein (%) CON: NR EX: NR ↔ Fat (%) CON: NR EX: NR ↔ CHO (%) CON: NR EX: NR ↔
			Pre-Intervention: Standardized breakfast Post-Intervention: Snack to replace exercise induced energy deficit as after-load Lunch buffet ad libitum 105 min post-exercise; dinner buffet ad libitum	CON: 1769 ± 532 EX: 1849 ± 486 ↔	Protein (%) CON: NR EX: NR ↔ Fat (%) CON: NR EX: NR ↔ CHO (%) CON: NR EX: NR ↔
Varley-Campbell et al. 2017	n: 38 (20 m, 18 f) Age: 13.0 ± 0.3 y BMI: 16.8 ± 2.2 kg/m^2^	Modality: Cycling Duration: until 1 MJ expended 31 to 56 min (44 ± 7 min) Intensity: moderate EE: 239 kcal	Pre-Intervention: Same breakfast on all experimental days, standardized snack in SK groups	CON: 1441 ± 113 (SE) CON + SK: 1367 ± 94 (SE) ↔	NR
			Post-Intervention: Lunch pizza meal ad libitum 65 min post- exercise	EX: 1496 ± 111 (SE) EX + SK: 1450 ± 103 (SE) ↔ SK = Snack (containing 239 kcal)	

*CON* Control group, *EX* Exercise group, *VO2max* maximal oxygen uptake, *VT* Ventilation threshold, *EE* Energy expenditure, *NR* Not reported, *SD* standard deviation, *SE* standard error

↑intake significantly higher

↓intake significantly lower

↔ no significant change

The Original Article has been corrected.

The Publisher apologizes for the inconvenience caused to the authors and readers.

## References

[1] Hahn H, Friedel M, Niessner C, et al. Impact of physical activity on caloric and macronutrient intake in children and adolescents: a systematic review and meta-analysis of randomized controlled trials. Int J Behav Nutr Phys Act. 2024;21:76. 10.1186/s12966-024-01620-8. 39010114 10.1186/s12966-024-01620-8 PMC11247817

