# Supplementary material for: Impact of physical activity on caloric and macronutrient intake in children and adolescents: a systematic review and meta-analysis of randomized controlled trials
Source: Int J Behav Nutr Phys Act. 2024 Jul 15;21:76. doi: 10.1186/s12966-024-01620-8 (PMC11247817; doi:10.1186/s12966-024-01620-8)
Supplement: Supplementary file 1 — Supplementary Material 1. [file 12966_2024_1620_MOESM1_ESM.docx]

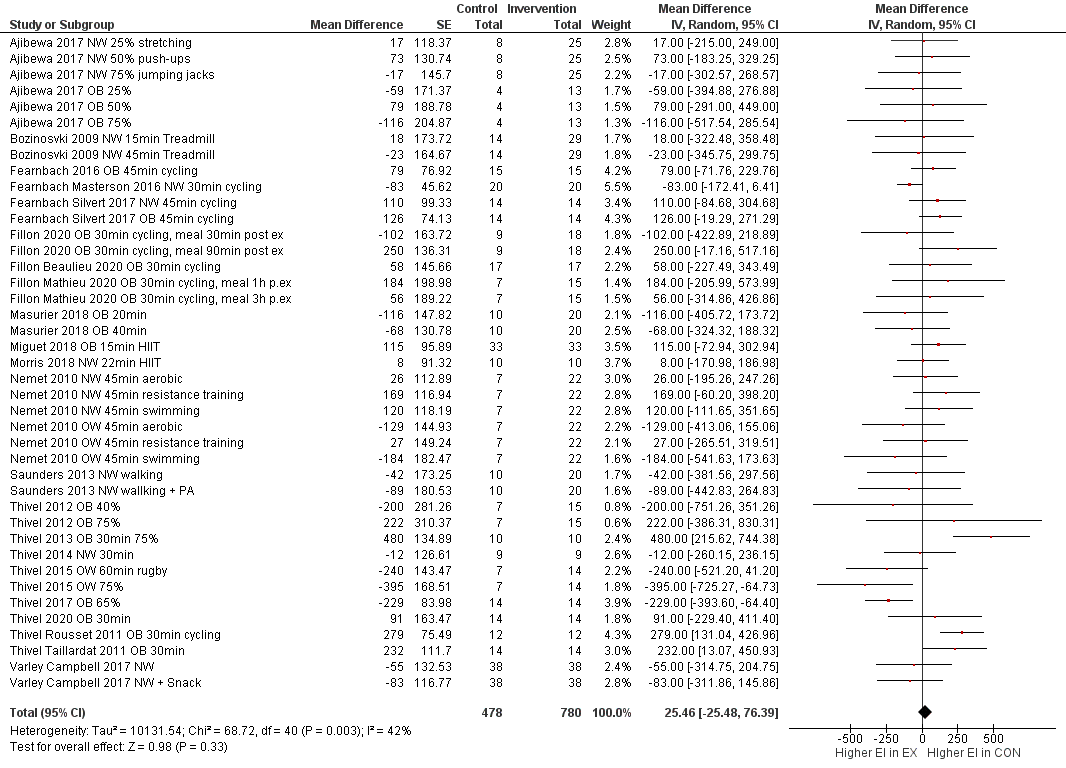


Figure 10 Sensitivity analysis for correlation coefficient = 0.3. Quantitative analysis for energy intake of randomized controlled trials. In multi-arm trials, the sample size of the shared control group was divided to prevent double counting.


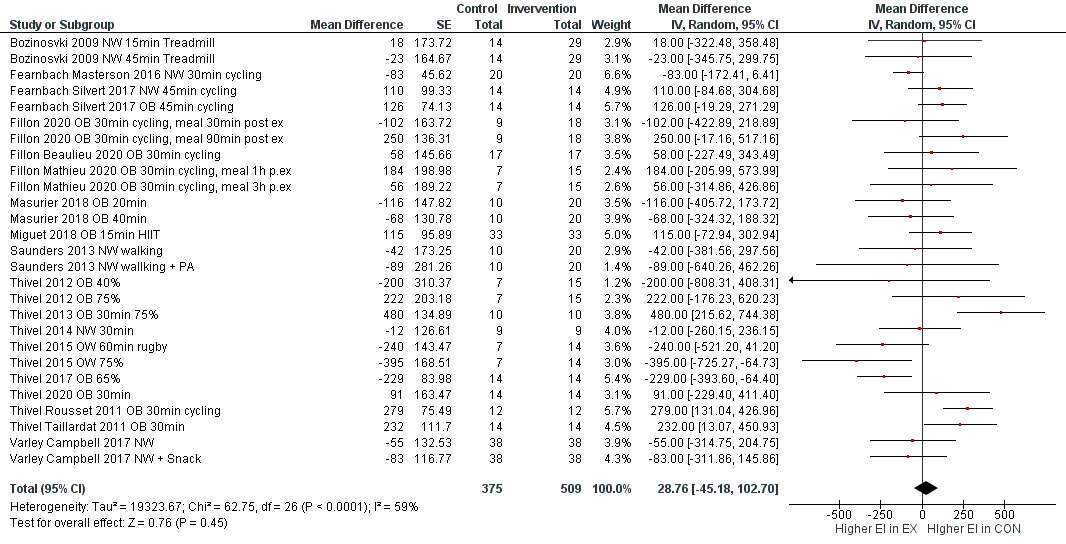


Figure 11 Sensitivity analysis for correlation coefficient = 0.3. Quantitative analysis for energy intake of randomized controlled trials with low or moderate risk of bias. In multi-arm trials, the sample size of the shared control group was divided to prevent double counting.


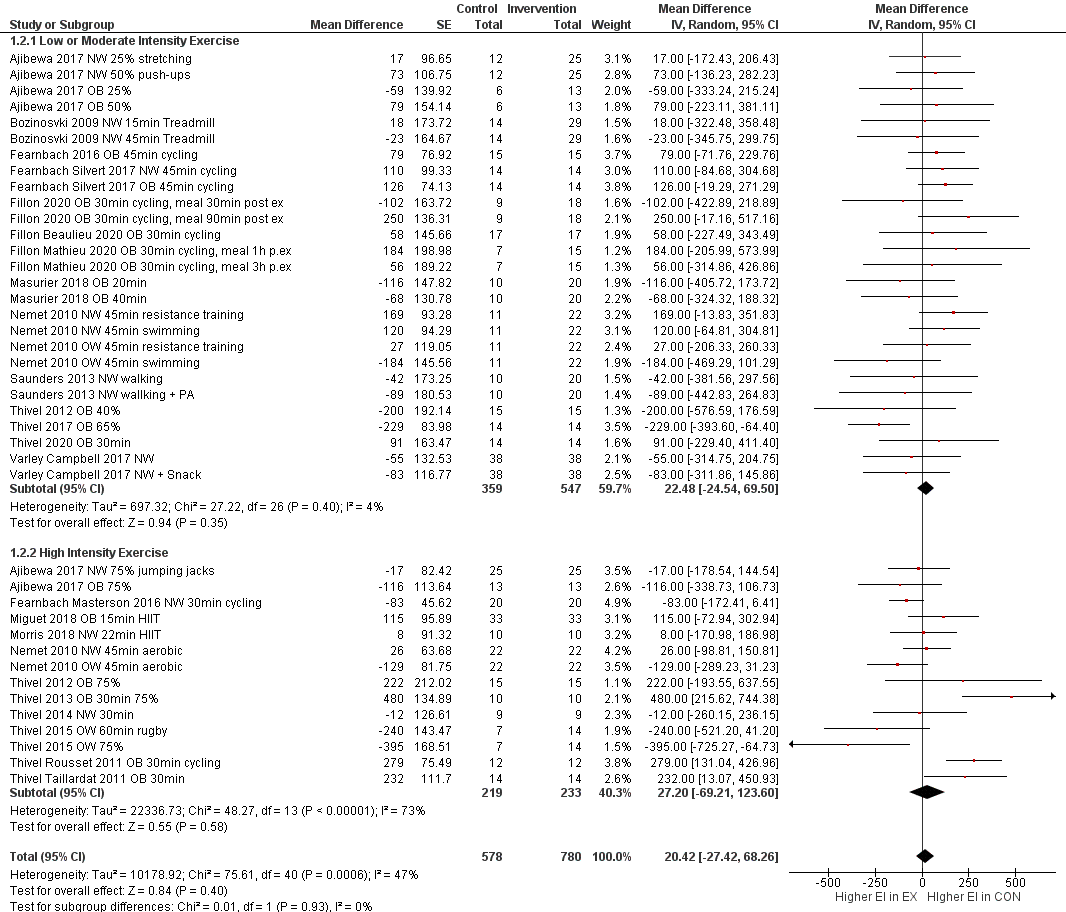


Figure 12 Sensitivity analysis for correlation coefficient = 0.3. Quantitative analysis for energy intake of randomized controlled trials categorized by low or moderate vs. high intensity exercise. In multi-arm trials, the sample size of the shared control group was divided to prevent double counting.


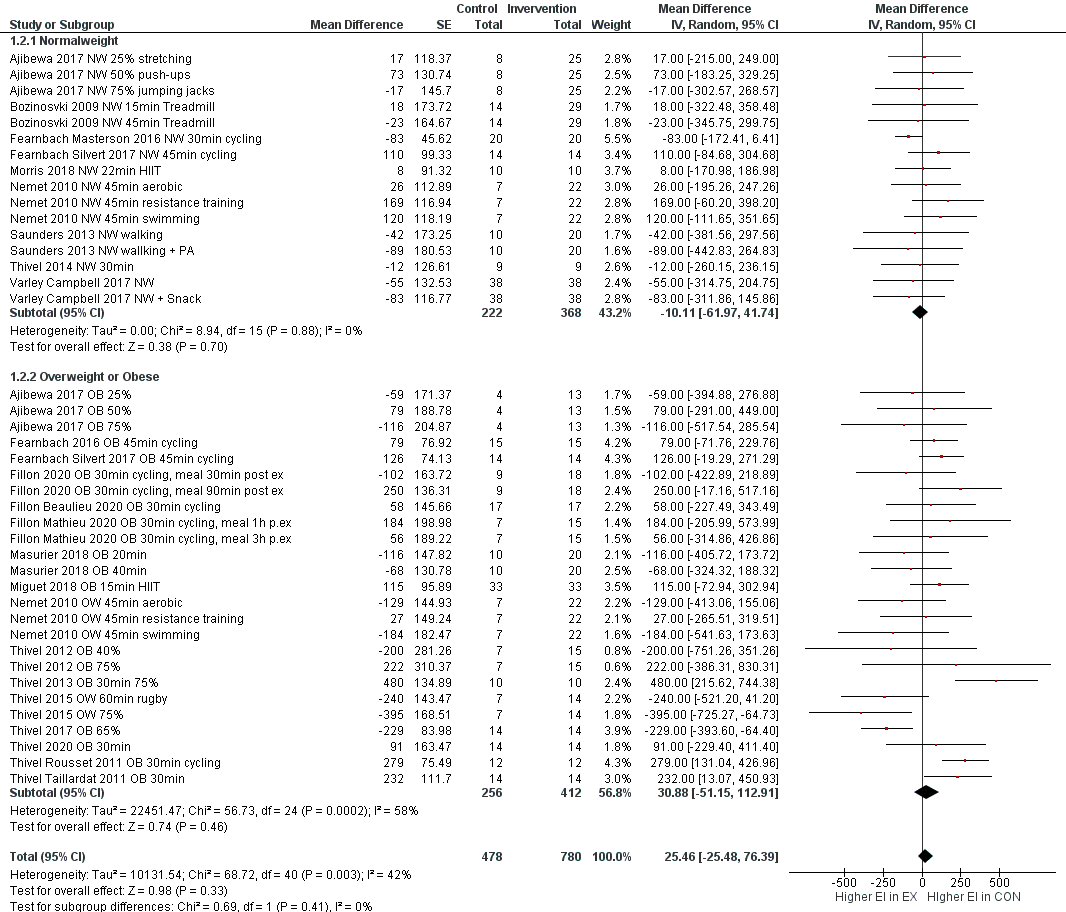


Figure 13 Sensitivity analysis for correlation coefficient = 0.3. Quantitative analysis for energy intake of randomized controlled trials categorized by subgroups with normal weight vs. overweight or obesity. In multi-arm trials, the sample size of the shared control group was divided to prevent double counting.


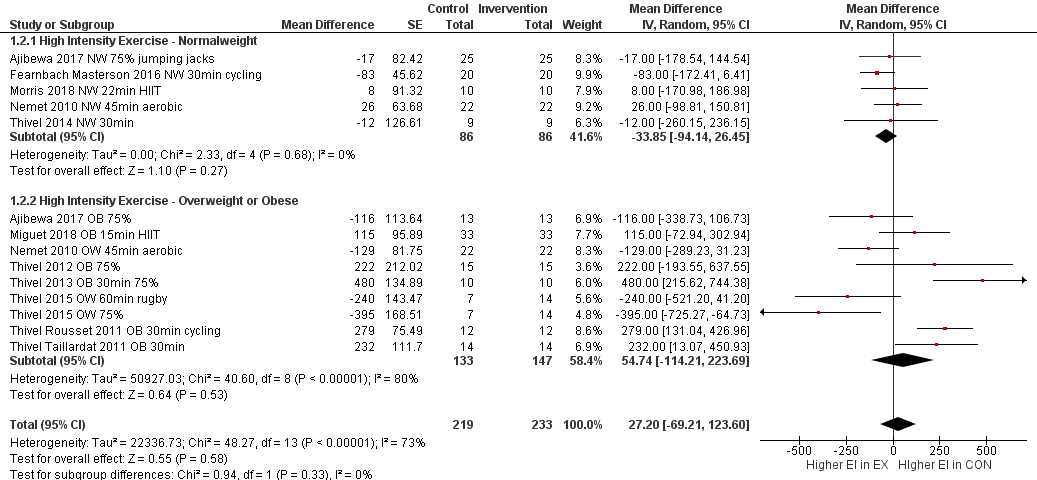


Figure 14 Sensitivity analysis for correlation coefficient = 0.3. Quantitative analysis for energy intake of randomized controlled trials with high intensity exercise categorized by subgroups with overweight or obesity vs. normal weight. In multi-arm trials, the sample size of the shared control group was divided to prevent double counting.


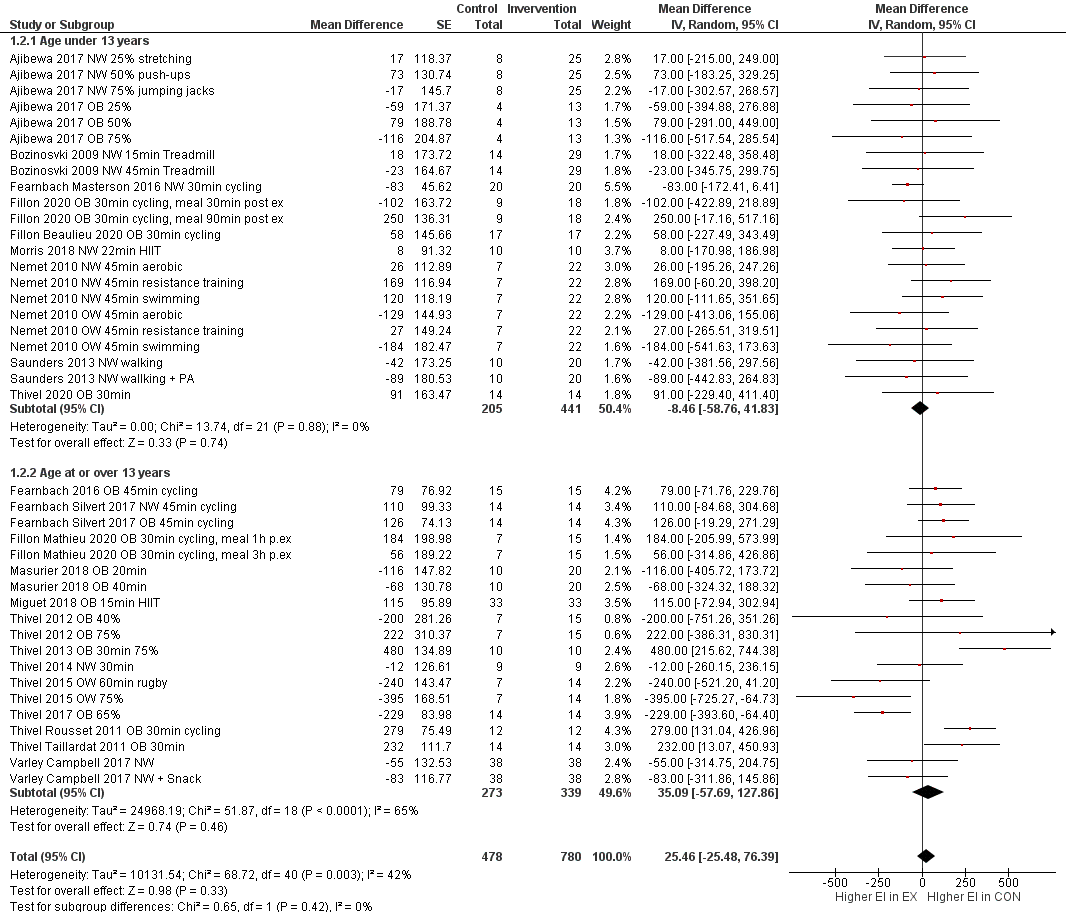


Figure 15 Sensitivity analysis for correlation coefficient = 0.3. Quantitative analysis for energy intake of randomized controlled trials for subgroups aged < 13 years vs. age ≥ 13 years. In multi-arm trials, the sample size of the shared control group was divided to prevent double counting.


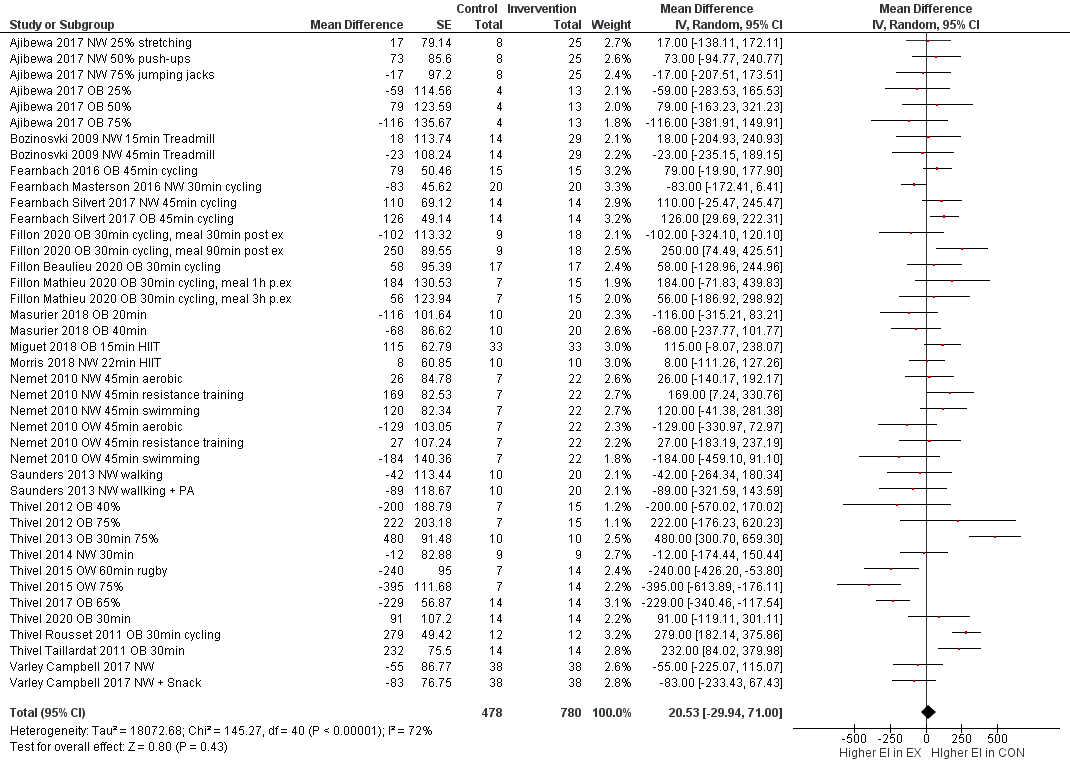


Figure 16 Sensitivity analysis for correlation coefficient = 0.7. Quantitative analysis for energy intake of randomized controlled trials. In multi-arm trials, the sample size of the shared control group was divided to prevent double counting.


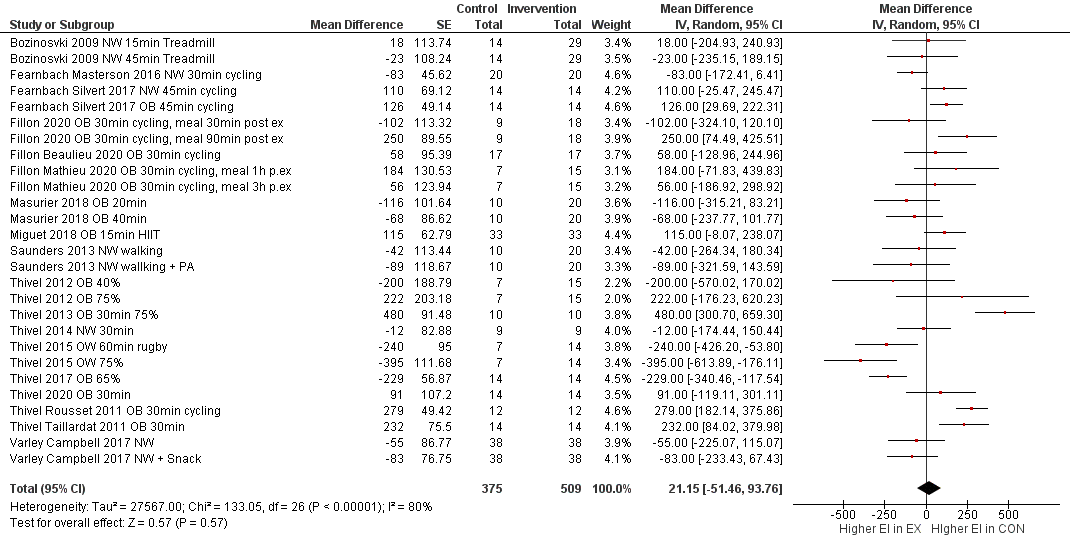


Figure 17 Sensitivity analysis for correlation coefficient = 0.7. Quantitative analysis for energy intake of randomized controlled trials with low or moderate risk of bias. In multi-arm trials, the sample size of the shared control group was divided to prevent double counting.


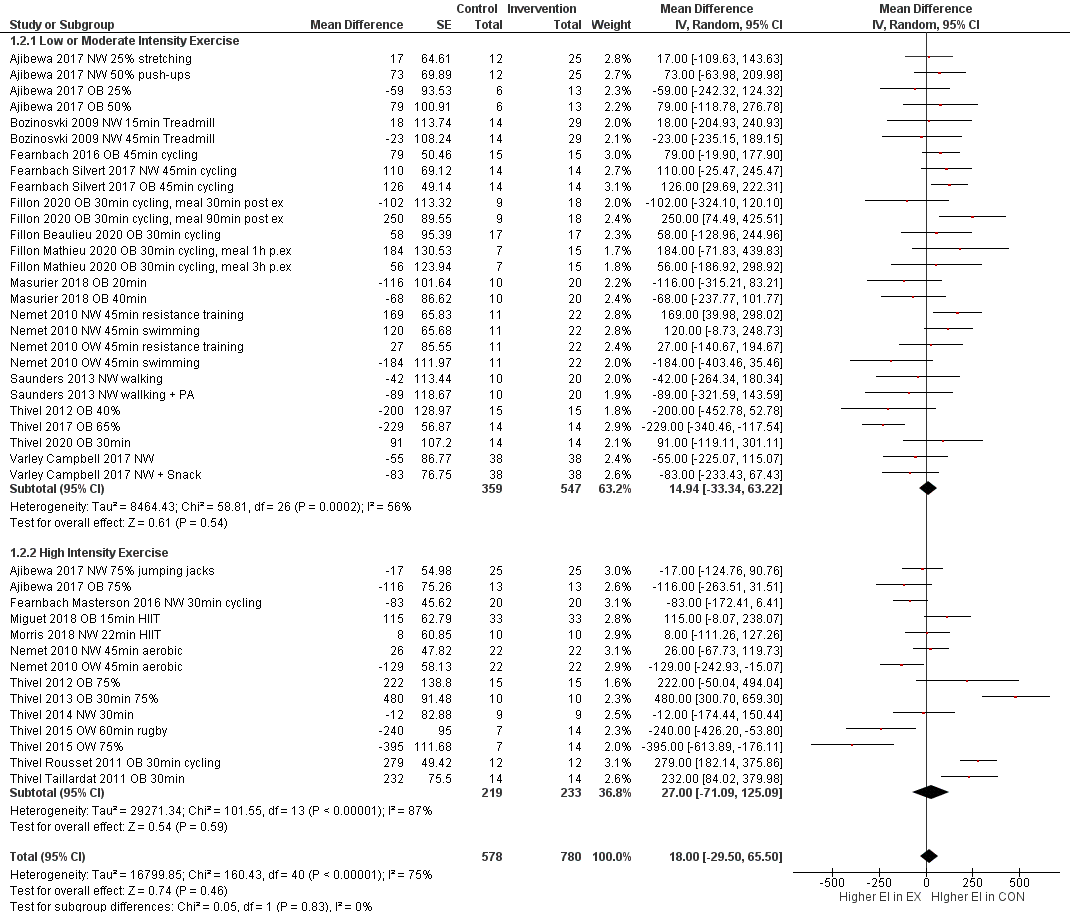


Figure 18 Sensitivity analysis for correlation coefficient = 0.7. Quantitative analysis for energy intake of randomized controlled trials categorized by low or moderate vs. high intensity exercise. In multi-arm trials, the sample size of the shared control group was divided to prevent double counting.


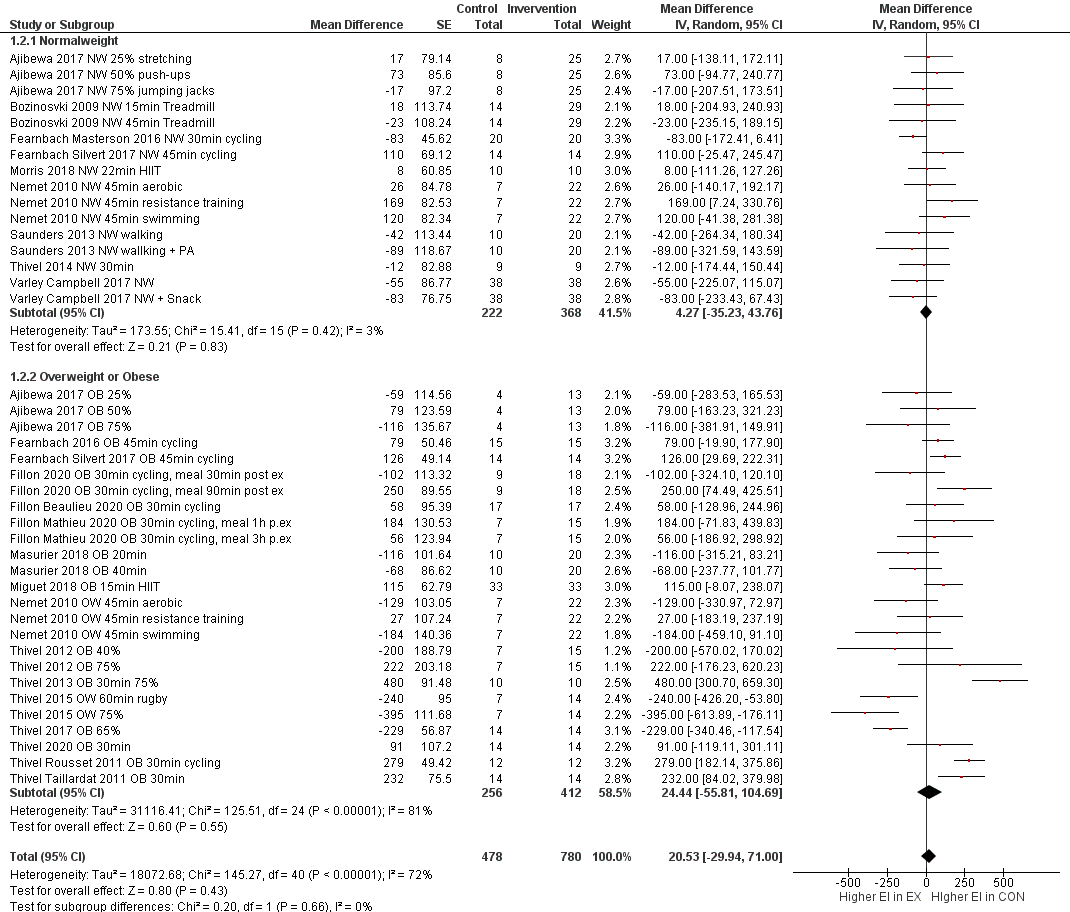


Figure 19 Sensitivity analysis for correlation coefficient = 0.3. Quantitative analysis for energy intake of randomized controlled trials categorized by subgroups with normal weight vs. overweight or obesity. In multi-arm trials, the sample size of the shared control group was divided to prevent double counting.


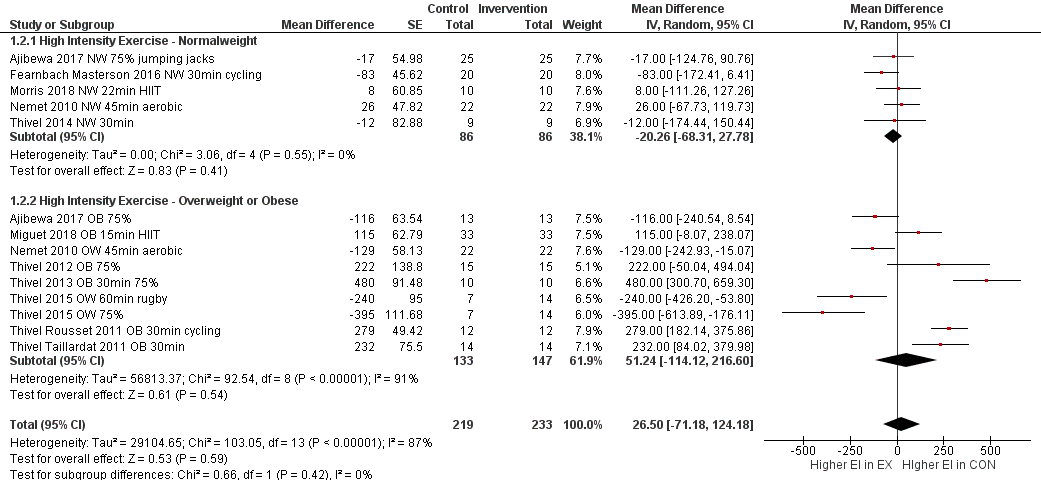


Figure 20 Sensitivity analysis for correlation coefficient = 0.7. Quantitative analysis for energy intake of randomized controlled trials with high intensity exercise categorized by subgroups with overweight or obesity vs. normal weight. In multi-arm trials, the sample size of the shared control group was divided to prevent double counting.


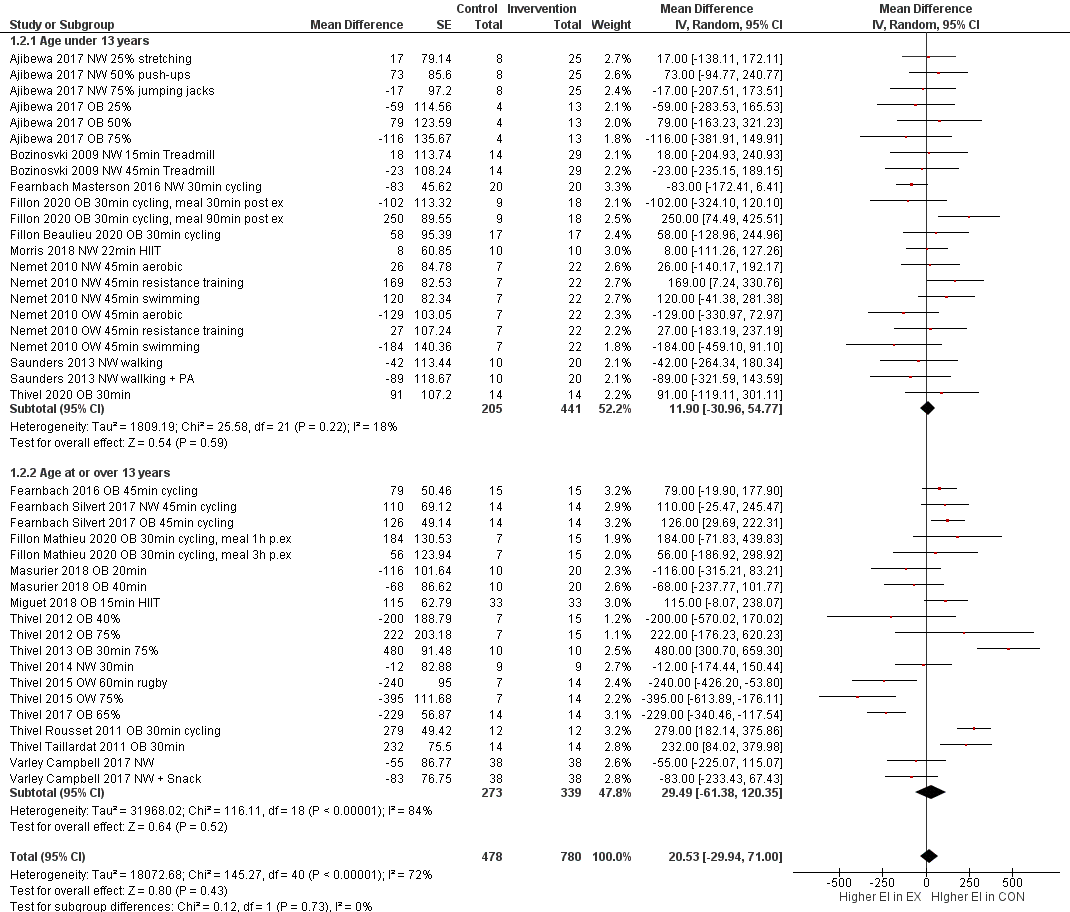


Figure 21 Sensitivity analysis for correlation coefficient = 0.7. Quantitative analysis for energy intake of randomized controlled trials for subgroups aged < 13 years vs. age ≥ 13 years. In multi-arm trials, the sample size of the shared control group was divided to prevent double counting.
