# Supplementary material for: Impact of physical activity on caloric and macronutrient intake in children and adolescents: a systematic review and meta-analysis of randomized controlled trials
Source: Int J Behav Nutr Phys Act. 2024 Jul 15;21:76. doi: 10.1186/s12966-024-01620-8 (PMC11247817; doi:10.1186/s12966-024-01620-8)
Supplement: Supplementary file 2 — Supplementary Material 2. [file 12966_2024_1620_MOESM2_ESM.docx]

**Impact of physical activity on caloric and macronutrient intake in children and adolescents: A Systematic Review and Meta-Analysis of randomized controlled trials**

Heiko Hahn^1^, Manuel Friedel^1^, Claudia Niessner^2^, Stephan Zipfel^1^, Isabelle Mack^1*^

^1^ Department of Psychosomatic Medicine and Psychotherapy, University Medical Hospital, Tübingen, Germany; ^2^ Institute of Sports and Sport Science, Karlsruhe Institute of Technology, 76131 Karlsruhe, Germany.

*****Correspondence: Isabelle Mack, University Medical Hospital Tübingen, Osianderstr. 5, 72076 Tübingen, Germany; E-Mail: isabelle.mack@uni-tuebingen.de

SUPPORTING INFORMATION:

Text S1. Search Strategy.

PubMed: ((((("Adolescent"[MeSH Terms] OR "Pediatrics"[MeSH Terms:noexp]) AND "Child"[MeSH Terms]) OR "girl*"[Title/Abstract] OR "Boys"[Title/Abstract] OR "child*"[Title/Abstract] OR "adolescen*"[Title/Abstract] OR "pediatric*"[Title/Abstract] OR "paediatric*"[Title/Abstract] OR "teen*"[Title/Abstract] OR "Kids"[Title/Abstract] OR "Kid"[Title/Abstract]) NOT ("adult"[MeSH Terms] NOT ("Adolescent"[MeSH Terms] OR "Child"[MeSH Terms] OR "child*"[Title/Abstract] OR "adolescen*"[Title/Abstract] OR "pediatric*"[Title/Abstract] OR "paediatric*"[Title/Abstract] OR "teen*"[Title/Abstract] OR "Kids"[Title/Abstract] OR "Kid"[Title/Abstract]))) AND ((("Sports"[MeSH Major Topic] OR "sport*"[Title/Abstract]) NOT "sports beverage*"[Title/Abstract]) OR "motor abil*"[Title/Abstract] OR "motor performance"[Title/Abstract] OR "Physical Fitness"[Title/Abstract] OR "physical activity"[Title/Abstract] OR "Exercise"[MeSH Major Topic] OR "VO2max"[Title/Abstract] OR "agil*"[Title/Abstract] OR (("activity program*"[Title/Abstract] OR "flexibility*"[Title/Abstract] OR "speed*"[Title/Abstract] OR "endurance*"[Title/Abstract] OR "coordination*"[Title/Abstract]) AND "Exercise"[Title/Abstract]) OR "Physical Fitness"[MeSH Terms]) AND ("Energy Intake"[MeSH Major Topic] OR "Energy Intake"[Title/Abstract] OR "Caloric Intake"[Title/Abstract] OR "calorie intake"[Title/Abstract] OR "eating/physiology"[MeSH Major Topic] OR "Food Intake"[Title/Abstract] OR "Diet"[MeSH Terms:noexp] OR "diet*"[Title/Abstract] OR "nutri*"[Title/Abstract] OR "macronutrient composition*"[Title/Abstract] OR "food composition*"[Title/Abstract] OR "nutrient composition*"[Title/Abstract] OR "food setting*"[Title/Abstract]) AND (("randomized controlled trial"[Publication Type] OR "randomized controlled trial*"[Title/Abstract] OR ("Randomized"[Title/Abstract] OR "Randomly"[Title/Abstract] OR "Epidemiologic Studies"[MeSH Terms] OR ("epidemiologic*"[Title/Abstract] AND "stud*"[Title/Abstract]) OR "longitudinal stud*"[Title/Abstract] OR "Cross-Sectional Studies"[MeSH Terms])) NOT ("Animals"[MeSH Terms] NOT "Humans"[MeSH Terms])))

Cochrane Wiley: ((pediatric* OR paediatric* OR adolescen* OR child* OR girl* OR boy* OR teen* OR kid OR kids):ti,ab,kw) NOT ([mh "adult"] NOT ([mh "adolescent"] OR [mh "pediatrics"] OR [mh "child"] OR (pediatric* OR adolescen* OR child* OR girl* OR boy* OR teen* OR paediatric* OR kid OR kids):ti,ab,kw)) ((motor* near/2 performance) OR (physical near/2 (fitness OR activit*)) OR exercise* OR VO2max OR agil*):ti,ab,kw ((energy OR caloric OR calorie OR food) near/2 intake) OR diet* OR nutri* OR ((macronutrient OR food OR nutrient) near/2 composition*) OR (food NEXT setting)):ti,ab,kw (Randomized Controlled Trial):pt OR (((randomized near/0 controlled near/0 trial*):ti,ab,kw) OR ((epidemiologic* OR longitudinal OR cross-sectional) near/0 (studies OR study))) NOT ([mh "animals"] NOT [mh "humans"])

Web of Science: TS=(adolesen* OR pediatric* OR paediatric* OR child* OR girl$ OR boy$ OR teen* OR kid$) NOT (TS=(adult$ NOT (adolesen* OR pediatric* OR paediatric* OR child* OR girl$ OR boy$ OR teen* OR kid$))) TS=(adipos* OR obes* OR overweight) TI=(sport* OR Exercise$) OR AB=(sport* OR Exercise$) OR TS=((motor NEAR/2 (abil* OR performance$)) OR (physical NEAR/2 (fitness OR activity)) OR VO2max OR agil* OR ((“activity program$” OR flexibility* OR speed* OR edurance$ OR coordination) NEAR/5 Exercise$)) TI=(((Energy OR Food OR caloric) NEAR/0 intake) OR diet* OR (eat* NEAR/5 physiolog*)) OR AB=(((Energy OR Food OR caloric) NEAR/0 intake) OR diet* OR (eat* NEAR/5 physiolog*)) OR TS=(((macronutr* OR food OR nutr*) NEAR/2 composition$) OR (food NEAR/2 setting$)) TS=((randomized NEAR/0 controlled NEAR/0 trial$) OR RCT OR ((epidemiolog* OR longitudinal* OR cross-sectional*) NEAR/2 (study OR studies))) NOT TS=(animal$ NOT human$)
